# Electronic anisotropy and rotational symmetry breaking at a Weyl semimetal/spin ice interface

**DOI:** 10.1126/sciadv.adr6202

**Published:** 2025-06-13

**Authors:** Tsung-Chi Wu, Yueqing Chang, Ang-Kun Wu, Michael Terilli, Fangdi Wen, Mikhail Kareev, Eun Sang Choi, David Graf, Qinghua Zhang, Lin Gu, Zhentao Wang, Jedediah H. Pixley, Jak Chakhalian

**Affiliations:** ^1^ Department of Physics and Astronomy, Rutgers University, Piscataway, NJ 08854, USA.; ^2^ Center for Materials Theory, Rutgers University, Piscataway, NJ 08854, USA.; ^3^ National High Magnetic Field Laboratory, Tallahassee, FL 32310, USA.; ^4^ Beijing National Laboratory for Condensed Matter Physics, Institute of Physics, Chinese Academy of Sciences, Beijing 100190, China.; ^5^ Beijing National Center for Electron Microscopy and Laboratory of Advanced Materials, Department of Materials Science and Engineering, Tsinghua University, Beijing 100084, China.; ^6^ Center for Correlated Matter and School of Physics, Zhejiang University, Hangzhou 310058, China.; ^7^ Center for Computational Quantum Physics, Flatiron Institute, New York, NY 10010, USA.

## Abstract

In magnetic pyrochlore materials, the interplay of spin-orbit coupling, electronic correlations, and geometrical frustration gives rise to exotic quantum phases, including topological semimetals and spin ice. While these phases have been observed in isolation, the interface-driven phenomena emerging from their interaction have never been realized previously. Here, we report on the discovery of interfacial electronic anisotropy and rotational symmetry breaking at a heterostructure consisting of the Weyl semimetal Eu_2_Ir_2_O_7_ and spin ice Dy_2_Ti_2_O_7_. Subjected to magnetic fields, we unveil a sixfold anisotropic transport response that is theoretically accounted by a Kondo-coupled heterointerface, where the spin ice’s field-tuned magnetism induces electron scattering in the Weyl semimetal’s topological Fermi-arc states. Furthermore, at elevated magnetic fields, we reveal a twofold anisotropic response indicative of the emergence of a symmetry-broken many-body state. This discovery showcases the potential of pyrochlore frustrated magnet/topological semimetal heterostructures in search of emergent interfacial phenomena.

## Teaser

When two exotic quantum matters, Weyl semimetal and spin ice, meet at an interface, they interact, leading to emergent phenomena.

## INTRODUCTION

The interface between two distinct quantum materials offers the rare opportunity to couple seemingly unrelated many-body ground states to create exotic phases that would otherwise be impossible (*1*–*4*). While there has been a plethora of novel strongly interacting phases discovered in stacked weakly correlated materials, such as graphene- and transition metal dichalcogenide–based heterostructures (*5*–*16*), constructing interfaces out of strongly correlated materials, whose individual layers already hold a great deal of intriguing phenomena, is also expected to have substantial promise (*17*–*20*).

Among the diverse set of strongly correlated materials that show promise for exploring interface-driven exotic phases, the magnetic pyrochlore materials (A_2_B_2_O_7_, with A being rare earth and B being Ir or Ti) stand out as particularly hopeful candidates. Here, the many-body phases arise from the interplay of topology, electronic correlations, and magnetic frustration due to their large spin-orbit coupling and unique corner-sharing tetrahedron lattice structure (*21*, *22*). Moreover, the strengths of the electronic correlations and spin-orbit coupling can be tuned by choosing different A and B ions, generating a rich phase diagram in magnetic pyrochlores (*21*–*23*). It is thus intriguing to devise interfacial quantum states through the thin-film heterostructure assembly of magnetic pyrochlores with inherently different many-body states (*24*–*27*).

While creating such high-quality interfaces remains a notable experimental challenge, two material classes show promising potential. The first is the pyrochlore iridates, A_2_Ir_2_O_7_ (A = lanthanide ions), that can harbor an antiferromagnetic Weyl semimetal phase with strongly spin-orbit coupled Ir, providing itinerant pseudo-relativistic electrons (*20*, *23*, *28*). The second is the pyrochlore titanates spin ice compounds, X_2_Ti_2_O_7_ (X = Ho and Dy), that are insulating frustrated magnets with magnetic excitations governed by strong dipolar interactions (*29*–*34*). The synthesis of pristine pyrochlore heterostructures has historically been highly challenging until very recent advances in creating high-quality heterojunction of pyrochlore iridates and titanates using a newly developed hybrid in situ solid-state epitaxy method (*35*). Such advancement enables the exploration of interfacial phenomena through the coupling of topological Fermi arcs inherent to the Weyl semimetal with the magnetic excitations characteristic of the spin ice (*20*, *36*, *37*). Toward this notion, we create a high-quality pyrochlore heterostructure, Eu_2_Ir_2_O_7_/Dy_2_Ti_2_O_7_ (EIO/DTO), which has a pristine oriented interface between a Weyl semimetal and classical spin ice. The synthetic structure exhibits a sixfold anisotropic response in angular-resolved magnetotransport upon application of an external magnetic field at ultralow temperatures. Such surprising behavior is linked to the Kondo-coupled magnetic states of the spin ice DTO, which induce electron scattering in the topological Fermi-arc states of the Weyl semimetal EIO. At higher magnetic fields, the system transitions into a rotational symmetry-broken twofold anisotropic state. This discovery highlights the potential of frustrated magnet/topological semimetal heterostructures for realizing unique many-body states by coupling exotic magnetic excitations with topologically nontrivial surface states.

## RESULTS

### Crystal structure and temperature dependence of longitudinal resistivity

**Fig. 1.**
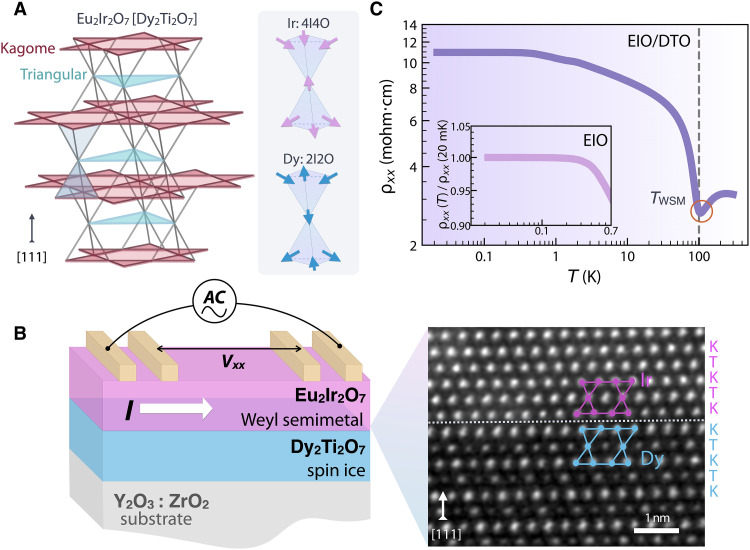
Crystal structure and temperature dependence of resistivity of EIO/DTO. (**A**) (Left) Crystal structure of the pyrochlore materials EIO (DTO) viewed along the [111] orientation, whose cations form interpenetrating tetrahedrons. Along the [111] direction, the sublattices of ions feature an alternating stacking of triangular and kagome planes. Oxygen sublattices are not shown. (Right) The Ir sublattice in EIO and the Dy sublattice in DTO show four-in-four-out (4I4O) and two-in-two-out (2I2O) spin structures, respectively. (**B**) Schematic experimental setup for electrical transport measurements on the pyrochlore heterostructure, EIO/DTO, consisting of [111]-oriented thin films of EIO and DTO that are Weyl semimetal and spin ice, respectively. EIO/DTO is grown on a nonmagnetic insulating substrate, YSZ. The current is applied along the [$1\bar{1}0$] direction. The zoom-out view shows the real-space image near the interface using scanning tunneling electron microscopy. The corresponding Ir and Dy ions are shown in pink and blue colors. K and T denote kagome and triangular layers, respectively. (**C**) Temperature-dependent resistivity $\rho_{\mathrm{xx}}\left(T\right)$ of EIO/DTO, showing an upturn at $T_{\text{WSM}}$ and a plateau at low T. The inset shows the temperature-dependent resistivity data of the control sample, EIO thin films grown on YSZ (denoted as EIO), with $\rho_{\mathrm{xx}}\left(T\right)/\rho_{\mathrm{xx}}$ (20 mK), from 20 to 700 mK, where a plateau region occurs at T ≤ 0.3 K.

Both EIO and DTO compounds have the identical nested corner-sharing tetrahedra of the cations and the global cubic crystal symmetry $\mathrm{Fd}\bar{3}m$. As illustrated in Fig. 1A, when viewed along the [111] direction, each cation sublattice forms an alternating stacking of triangular and kagome planes. Although bulk pyrochlore iridates were one of the first material candidates predicted to host the magnetic Weyl semimetal phase (*23*, *28*, *38*), the experimental identification of the phase had been elusive due to the zero anomalous Hall effect (AHE) enforced by the cubic symmetry. Recent theoretical and experimental findings, however, have demonstrated that films of pyrochlore iridates grown along the [111] direction manifest a pronounced AHE arising from the presence of Weyl nodes, confirming the topologically nontrivial nature of EIO (*37*, *39*).

For this study, we synthesized a high-quality heterostructure composed of EIO and DTO layers along the [111] orientation of the pyrochlore lattice on the insulating substrate Y_2_O_3_:ZrO_2_ (YSZ) (Fig. 1B). Notice that the [111] oriented EIO thin film grown directly on the YSZ substrate (EIO) is referred to as the control sample. At low temperatures, EIO shows the antiferromagnetic Weyl semimetal state with a four-in-four-out (4I4O/4O4I) long-range order of the Ir moments (*28*, *37*, *39*, *40*), while DTO exhibits the magnetically degenerate spin ice phase with the imposed two-in-two-out (2I2O/2O2I) spin ice rule on the Dy moments (Fig. 1A) (*29*–*33*). Here is an intriguing question: Can passing electrical currents across the EIO/DTO interface transpose the magnetic excitations of the insulating spin ice DTO (Fig. 1B) into the electronic degrees of freedom of the conducting Weyl semimetal EIO?

Figure 1C shows the temperature-dependent longitudinal resistivity $\rho_{\mathrm{xx}}\left(T\right)$ of EIO/DTO with the current (I) applied along the [$1\bar{1}0$] direction. Several characteristic features are immediately seen. First, when cooling down below 300 K, a metal-to-semimetal–like transition occurs at $T_{\text{WSM}}$ ≈ 105 K, consistent with the previously reported magnetic phase transition of thin-film EIO from a paramagnetic metal phase to an antiferromagnetic Weyl semimetal phase (*37*). Second, upon further decreasing temperatures below 0.3 K, $\rho_{\mathrm{xx}}\left(T\right)$ develops a plateau. The measurement on the control sample reveals the presence of both the WSM transition near $T_{\text{WSM}}$ and the plateau region (see fig. S3 and inset in Fig. 1C). These results establish that the two transport signatures arise from the properties inherent in the EIO layer. Notably, the low-T plateau has been attributed to the presence of topological surface states that are more conductive than the bulk (*41*–*55*). For the Weyl semimetal EIO, these topological surface states consist of the Weyl Fermi-arc surface states (*28*, *56*, *57*).

### Angular dependence of magnetoresistance under out-of-plane field rotation

**Fig. 2.**
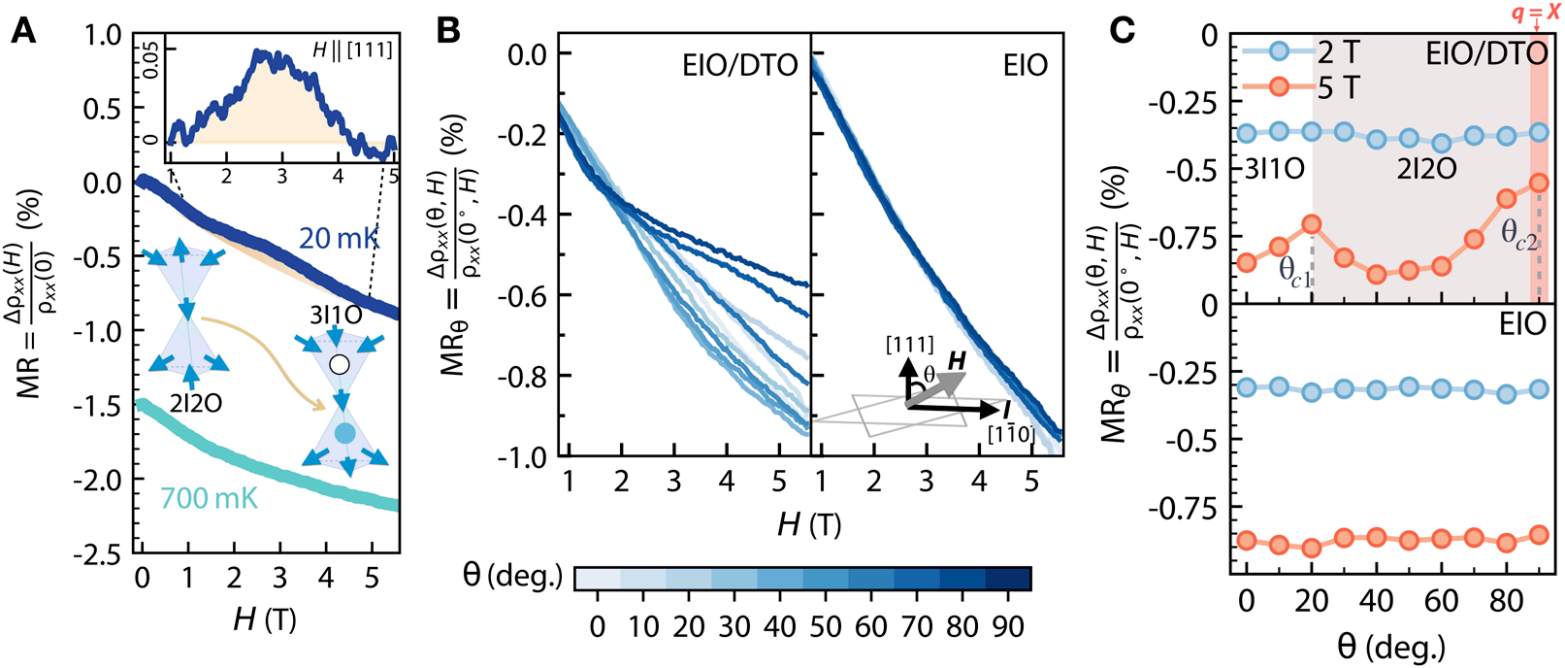
Angular dependence of MR for field rotation away from [111] direction in EIO/DTO. (**A**) MR of EIO/DTO with H ∥ [111]. An anomalous MR with a bump feature occurs at 20 mK (blue) (the enclosed yellow area). The inset shows the bump feature after subtracting the background of the raw data (see Supplementary Materials for the raw data). In contrast, no bump feature is found at 700 mK (cyan; offset −1.5% for clarity). The left and right tetrahedrons show that the spin structures change from a two-in-two-out (2I2O/2O2I) to a three-in-one-out (3I1O/1I3O) configuration. The latter is the so-called magnetic monopole state of DTO. The 3I1O and 1I3O spin structures host a monopole (white circle) and anti-monopole (blue circle), respectively. (**B**) ${\text{MR}}_{\theta }=\Delta \rho_{\mathrm{xx}}\left(\theta ,H\right)/\rho_{\mathrm{xx}}\left(0^{\circ },H\right)$ of EIO/DTO (left) and the control sample EIO (right) at 20 mK for different field angles, θ. The inset shows the rotational geometry of fields. (**C**) ${\text{MR}}_{\theta }=\Delta \rho_{\mathrm{xx}}\left(\theta ,H\right)/\rho_{\mathrm{xx}}\left(0^{\circ },H\right)$ of EIO/DTO (top) and the control sample EIO (bottom) at 2 and 5 T at $\theta =0^{\circ },10^{\circ },\ldots ,90^{\circ }$ [extracted from (B)]. $\theta_{c1}$ and $\theta_{c2}$ are defined as the two peak positions at 20° and 90° for H=5T, respectively. As θ increases from 0° to $\theta_{c1}$ and to $\theta_{c2}$, DTO changes from 3I1O to 2I2O and to q=X phases.

Now, we can investigate the interaction between the Weyl Fermi arcs and spin ice’s magnetism. For this purpose, we use magnetotransport measurements near the resistivity plateau region at sub-kelvin temperatures. Figure 2A shows the magnetoresistance (MR) of EIO/DTO taken at 20 mK under an applied magnetic field H ∥ [111], where $\text{MR}=\Delta \rho_{\mathrm{xx}}\left(H\right)/\rho_{\mathrm{xx}}\left(0\right)=\left[\rho_{\mathrm{xx}}\left(H\right)-\rho_{\mathrm{xx}}\left(0\right)\right]/\rho_{\mathrm{xx}}\left(0\right)$. As immediately seen, a bump-like feature develops between 1 and 5 T, residing on a background of the negative MR signal characteristic of Weyl semimetals (*58*, *59*). Moreover, the temperature dependence of the MR in the EIO/DTO heterostructure reveals that the amplitude of the MR feature diminishes with increasing temperatures and vanishes at approximately 700 mK (see Fig. 2A and fig. S6). In contrast, a careful examination of the control sample shows only the anticipated negative MR signal, without additional anomalies to 20 mK (see fig. S3). This stark distinction between the EIO/DTO heterostructure and the single EIO layer provides compelling evidence that the anomalous transport signature observed in EIO/DTO stems from a nontrivial coupling at the interface.

To deepen our understanding of the MR bump-like feature and provide further ground for the interpretation, we recap that in bulk DTO, when a magnetic field is aligned along the [111] direction, it triggers a phase transition that violates the ice rule, transitioning from a kagome ice phase with a two-in-one-out (2I1O/1O2I) configuration within the kagome planes to a magnetic monopole phase characterized by three-in-one-out (3I1O/1O3I) spin-dressed tetrahedra shown in Fig. 2A (*29*, *30*).

By comparing the magnetization (M) versus the magnetic field (H) between bulk DTO and the heterostructure EIO/DTO configuration, we can draw a direct connection between the observed MR anomaly in the EIO/DTO heterostructure with the ice rule–violating transition into the magnetic monopole phase (for detailed analysis, see section 1.6 of the Supplementary Text). This finding supports our conjectures that electric transport under an applied magnetic field acquires sufficient sensitivity to reveal the magnetic phase transition in DTO encoded in the dynamics of Weyl electrons across the EIO/DTO interface. Furthermore, since the magnetic states of DTO can be precisely manipulated through a choice of angles and strengths of the applied magnetic field, we can further probe the dynamics of the interfacial interactions between the Weyl semimetal and spin ice.

Figure 2B shows the angular dependence of the MR in EIO/DTO, defined as ${\text{MR}}_{\theta }=\Delta \rho_{\mathrm{xx}}\left(\theta ,H\right)/\rho_{\mathrm{xx}}\left(0^{\circ },H\right)=\left[\rho_{\mathrm{xx}}\left(\theta ,H\right)-\rho_{\mathrm{xx}}\left(0^{\circ },H\right)\right]/\rho_{\mathrm{xx}}\left(0^{\circ },H\right)$, with H rotating away from [111] to $\left[1\bar{1}0\right]$ direction as illustrated in Fig. 2B’s inset. As seen, above 2 T, we observe a strongly angle-dependent MR_θ_ in EIO/DTO, which is entirely absent in the control sample EIO. To further clarify the angular dependence, we plot the values of MR_θ_ at fixed field strengths of 2 and 5 T (Fig. 2C). A direct inspection of the results reveals that at 2 T, no discernible anisotropy is found for both the EIO/DTO and EIO. In contrast, at 5 T, two distinct peaks develop near $\theta_{c1}=20^{\circ }$ and $\theta_{c2}=90^{\circ }$ only in EIO/DTO.

A straightforward comparison of these observed features in the EIO/DTO with the reported magnetic phase transitions in bulk DTO (*27*, *29*–*33*) confirms that $\theta_{c1}$ aligns with the phase boundary transition from the 3I1O phase to the 2I2O phase, while $\theta_{c2}$ marks the transition from the 2I2O phase to the q=X magnetic phase characterized by antiferromagnetic spin chains oriented perpendicular to the applied field direction (also see fig. S13). These findings unambiguously demonstrate the remarkable sensitivity of Weyl fermions at the Fermi arcs of EIO to the spin dynamics of DTO at the EIO/DTO interface, rigorously modulated by the orientation and intensity of the applied magnetic field. Distinct transport responses are expected in heterostructures composed of DTO and ordinary metals (see section 1.8.3 of the Supplementary Text).

### Angular dependence of MR under in-plane field rotation

**Fig. 3.**
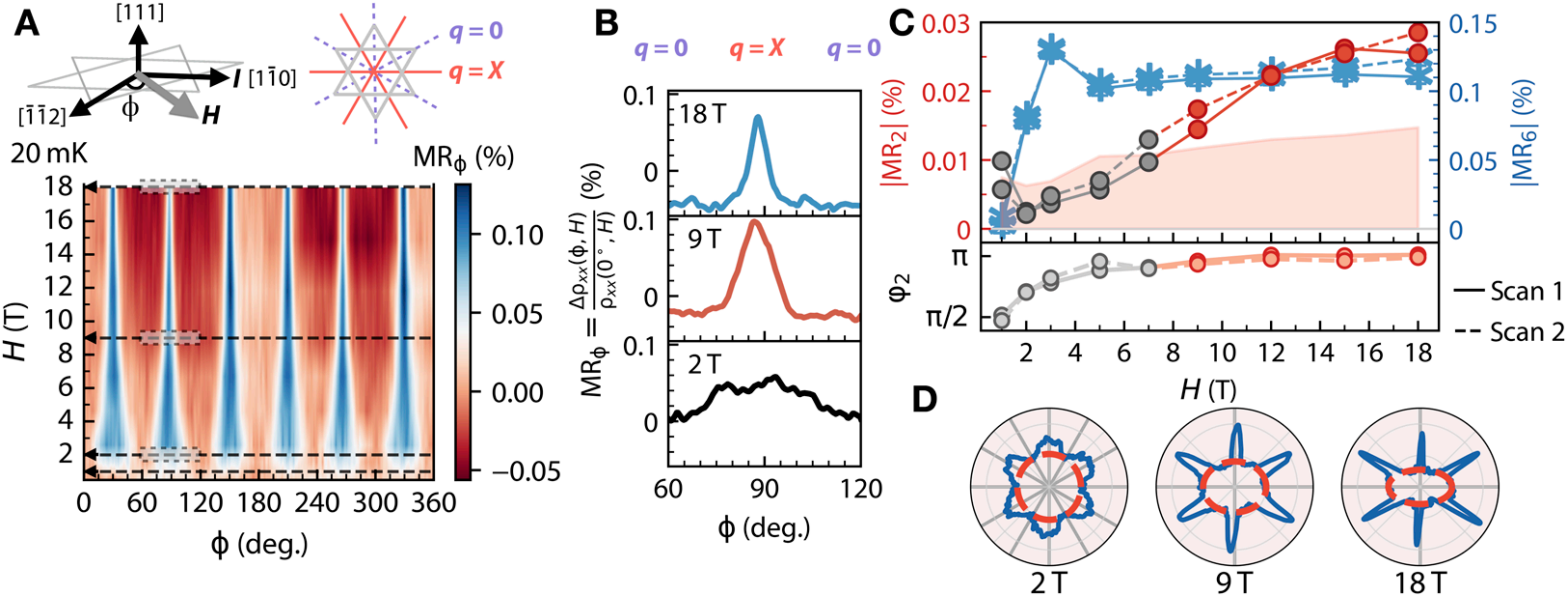
Angular dependence of MR for field rotation within (111) plane in EIO/DTO. (**A**) (Top) The left scheme shows the geometry for field rotation angles, ϕ. The right scheme shows q=0 (dash line) and q=X (solid line) phases of DTO happen at $\phi =60n^{\circ }$ and $\phi ={\left(60n+30\right)}^{\circ }$, respectively, where n = 0, 1, 2, 3, 4, and 5. (Bottom) Contour plot of ${\text{MR}}_{\phi }=\Delta \rho_{\mathrm{xx}}\left(\phi ,H\right)/\rho_{\mathrm{xx}}\left(0^{\circ },H\right)$ at 20 mK in EIO/DTO for H up to 18 T. (**B**) ${\text{MR}}_{\phi }=\Delta \rho_{\mathrm{xx}}\left(\phi ,H\right)/\rho_{\mathrm{xx}}\left(0^{\circ },H\right)$ at 20 mK for ϕ from 60° to 120° at 2 T (black), 9 T (red), and 18 T (blue). The narrowing of the MR peak at 90° is found while increasing magnetic fields. While the low MR values happen at 60° to 120° where DTO is in q=0 state, the high MR value happens at 90° where DTO is in the q=X state. (**C**) Magnitudes of the two- and sixfold contributions to ${\text{MR}}_{\phi }$, ${\text{MR}}_{2}$, and ${\text{MR}}_{6}$, with two datasets shown (scans 1 and 2). From 2 to 7 T, the magnitude of the twofold contribution $|{\text{MR}}_{2}|$ lies below the uncertainty of the fitting. For fields above 9 T, the twofold anisotropy is clearly visible and shifts in its phase by π/2 as shown in (C). On the other hand, the sixfold contribution $|{\text{MR}}_{6}|$ is present from 2 to 18 T. (**D**) Polar plots of ${\text{MR}}_{\phi }$ (of scan 2) at 20 mK at 2, 9, and 18 T, where the solid blue lines, solid gray lines, and red dashed lines denote the ${\text{MR}}_{\phi }$ data, mirror planes, and oval backgrounds, respectively. ${\text{MR}}_{\phi }$ shows a sixfold anisotropy at 2 T and a twofold anisotropy at 9 and 18 T.

**Fig. 4.**
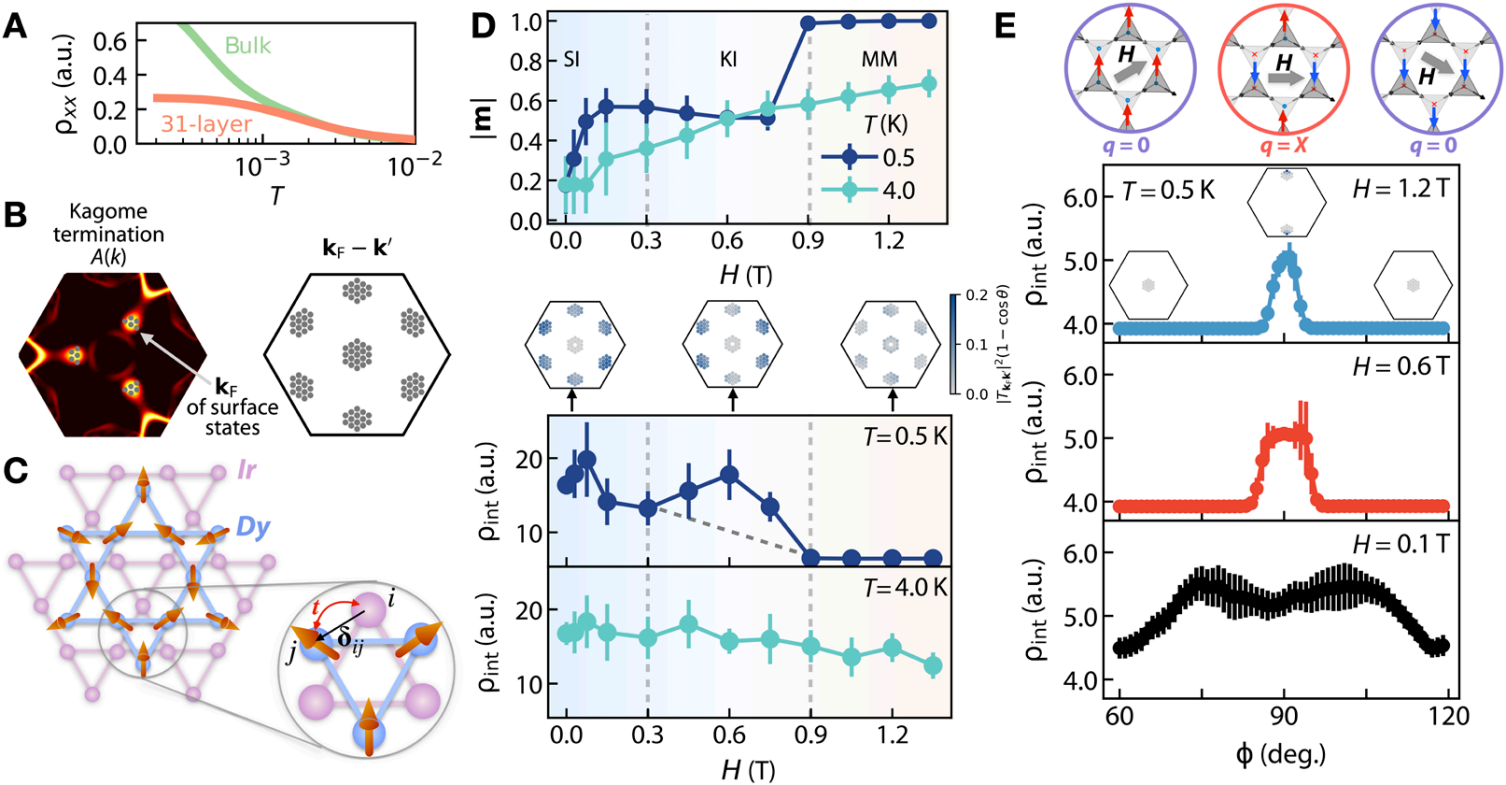
Phenomenological theoretical description of the observed transport behavior in EIO/DTO. (**A**) Theoretically computed $\rho_{\mathrm{xx}}$ versus temperature T in bulk EIO and 31-layer EIO slab. $\rho_{\mathrm{xx}}$ shows a saturated plateau at low-T in the slab but not in the bulk, demonstrating the effect of metallic surface states present in the slab. (**B**) Fermi arcs of the EIO slab at the kagome termination, where the Fermi vectors $k_{F}$ correspond to the three small electron pockets at the projected Weyl points. On the right, we show the momenta where the integration of $\tau_{\text{int}}^{-1}\left(k_{F}\right)$ is performed: $q=k_{F}-k\prime$, where $k\prime \in \left\{k_{F}\right\}$. (**C**) The interface of EIO and DTO realizes a Kondo coupling between the kagome Ir lattice and kagome Dy lattice. We include up to the nearest-neighbor Kondo coupling between the Ir and Dy orbitals i and j separated by vector $\delta_{\mathrm{ij}}$. (**D**) Upon increasing [111] magnetic field, the calculated magnetization of DTO first shows a crossover from the spin ice (SI) phase to the kagome ice (KI) phase, followed by an ice rule–breaking transition to the magnetic monopole (MM) phase (top). The computed interfacial resistivity $\rho_{\text{int}}$ shows a bump-like feature (bottom), consistent with the experiments (see Fig. 2A). The insets between two panels show the integral kernel of $\tau_{\text{int}}^{-1}\left(k_{F}\right)$, i.e., ${|T_{k_{F}k\prime }|}^{2}\left[1-\text{cos}\left(\theta \right)\right]$. (**E**) Computed $\rho_{\text{int}}$ versus ϕ, for $60^{\circ }\leq \phi \leq 120^{\circ }$ under in-plane magnetic fields H. At $\phi =60^{\circ },120^{\circ }$, $\rho_{\text{int}}$ is small as DTO shows q=0 phase, while at $\phi =90^{\circ }$, $\rho_{\text{int}}$ is large as DTO shows q=X phase. As fields increase, the peaks in $\rho_{\text{int}}$ become narrower, in agreement with the experiments (see Fig. 3B).

Having established the strong link between the Weyl fermions and specific spin ice magnetic configurations in EIO/DTO, we can now explore the microscopic characters of the interfacial states arising from such coupling. For this purpose, we turn to the angular-dependent MR measurement under in-plane applied magnetic fields at an angle ϕ, MR_ϕ_ (see Fig. 3A for the definition and geometry). A direct inspection of the data shown in Fig. 3B unveils several remarkable features. First, we identify a surprising sixfold rotational symmetric state that occurs after the magnetic field exceeds 2 T. Second, the transport signal shows a distinctive continuous angular narrowing at the specific $\phi_{n}=\left(30^{\circ }+60^{\circ }n\right)$ (n=0…5) directions where the MR reached its maxima. The lowest values of MR_ϕ_ are found in six $\phi_{n}$ directions along 60°n. To understand the sixfold anisotropic state at the interface between the EIO and DTO layers, we refer to Figs. 3A and 4E. Previous extensive investigations on single-crystal DTO (*60*–*63*) revealed that an in-plane magnetic field effectively controls the magnetic state of DTO by switching its magnetism from q=0 phase at $\phi_{n}=\left(60^{\circ }n\right)$ to q=X phase at $\phi_{n}=\left(30^{\circ }+60^{\circ }n\right)$. Remarkably, the appearance of the sixfold symmetric MR_ϕ_ is in excellent registry with the magnetic field–tuned cyclical switching between the q=0 and q=X spin structures of DTO. Furthermore, to shed light on the unusual angular narrowing, we plot MR_ϕ_ at 2, 9, and 18 T for ϕ varying from 60° to 120° (see Fig. 3B and the grayed area in Fig. 3A). By analyzing angular ${\text{MR}}_{\phi =90^{\circ }}$, which scales with $\Delta \rho_{\mathrm{xx}}\left(H\right)/\Delta \phi$, we reveal a gigantic almost 600% increase in the angular MR value at 18 T compared to that of 2 T (see fig. S7). Notably, the angular narrowing phenomenon is rare, closely resembling the large angular response reported in magnetic nodal crystals (*64*, *65*).

In addition to these two observations, another striking finding is the emergence of a quantum state when the applied magnetic field exceeds 9 T, as illustrated in Fig. 3B. At lower magnetic fields, just above 2 T, ${\text{MR}}_{\phi }$ exhibits six broad peaks against a uniform backdrop, implying a sixfold rotational symmetry. This symmetry transforms drastically once the magnetic field exceeds 9 T, where the ${\text{MR}}_{\phi }$ signal traverses into a twofold or bilateral symmetry state. Polar plots of the MR taken at 2, 9, and 18 T shown in Fig. 3C demonstrate this transition. At 2 T, the polar plot displays a sixfold rotational symmetry with an almost perfect circular background, consistent with the symmetry expected from the field-tuned q=0 and q=X magnetic structures. However, beyond 9 T, a distinct transition to a twofold symmetry with an oval-like background is observed. The degree of ovality also increases progressively up to 18 T (see fig. S18).

A comprehensive analysis of the MR signal (see section 1.9 of the Supplementary Text) reveals the evolution of two distinct contributions to the MR, namely, the sixfold $|{\text{MR}}_{6}|$ and the twofold $|{\text{MR}}_{2}|$ as a function of the magnetic field. While the $|{\text{MR}}_{6}|$ signal remains finite and nearly unchanged from 2 T to 18 T, the $|{\text{MR}}_{2}|$ signal appears right above 9 T and grows with increasing magnetic field strength up to 18 T. The absence of both field-induced features in MR_ϕ_ for the control sample emphasizes that the unique sixfold and twofold anisotropic states observed at H≥2 T and H≥9 T, respectively, are exclusively induced by the interface-coupled Weyl electrons of EIO and magnetic excitations of DTO (see fig. S3).

### Modeling of the Weyl semimetal/spin ice interface

In what follows, we describe a microscopic framework for understanding the observed magnetotransport phenomena in EIO/DTO, providing a qualitative explanation for the experimental findings (see section 1.8 of the Supplementary Text for more details). To achieve this, we devise an effective model for the heterostructure’s Hamiltonian, ${\hat{H}}_{\text{EIO}/\text{DTO}}={\hat{H}}_{\text{EIO}}+H_{\text{DTO}}+{\hat{H}}_{\text{int}}$, where the last term describes the interfacial interaction between the Dy spins and Ir states placed on the kagome lattice. We solve for ${\hat{H}}_{\text{EIO}}$ and $H_{\text{DTO}}$ separately before coupling them through a perturbative treatment of ${\hat{H}}_{\text{int}}$. First, we establish that EIO’s Weyl Fermi arcs expressed in ${\hat{H}}_{\text{EIO}}$ can give rise to a resistivity plateau at low temperatures. As shown in Fig. 4A, the computed $\rho_{\mathrm{xx}}\left(T\right)$ for EIO clearly reproduces the experimentally found low-temperature plateau in longitudinal resistance $\rho_{\mathrm{xx}}\left(T\right)$, which occurs only in the EIO slab but not in bulk EIO, thus attributing the plateau to the presence of the Weyl Fermi-arc surface states shown in the spectral function A(k) projected on a kagome terminated atomic plane in Fig. 4B.

To capture the physics encoded in the $H_{\text{DTO}}$ term, we use a dipolar classical spin model solved with a classical Monte Carlo (MC) approach at low temperatures. On the basis of those approaches, we accurately reproduce the reported M-H curve for the bulk kagome ice and magnetic monopole phases under a [111] applied magnetic field shown in Fig. 4D (top) (also see section 1.8.2 of the Supplementary Text and fig. S6), as well as the establishment of the quasi–one-dimensional magnetic structure under differently oriented magnetic fields (*66*) (also see section 1.8.2 of the Supplementary Text and fig. S19).

Next, we consider the interfacial interaction dominated by the Kondo coupling between the Fermi-arc surface states and the local Dy moments. As pictured in Fig. 4C, this coupling is represented by ${\hat{H}}_{\text{int}}=\sum_{\langle i,j\rangle }J_{\mathrm{ij}}S_{i}\cdot {\hat{s}}_{j}$, where $J_{\mathrm{ij}}$ is the interfacial superexchange between Dy (classical moments $S_{i}$) and Ir (quantum moments ${\hat{s}}_{j}$), i.e., Kondo coupling, and the sum is over the nearest neighbors between the two interfacial layers. Within the Boltzmann approximation (*67*), the coupling term introduces an interfacial resistance, $\rho_{\text{int}}$, by inducing a finite relaxation time for the conducting Fermi-arc surface states, i.e., $\rho_{\text{int}}\propto \tau^{-1}\left(k_{F}\right)\propto \int dk\prime {|T_{k_{F}k\prime }|}^{2}\delta \left(\epsilon_{F}-\epsilon_{k\prime }\right)\left(1-\text{cos}\theta_{k_{F}k\prime }\right)$, where $\epsilon_{k}$ is the dispersion of the surface states of EIO, and $\theta_{k_{F}k\prime }$ is the scattering angle. The scattering matrix element ${|T_{k_{F}k\prime }|}^{2}$ is proportional to the DTO’s spin structure factor $S\left(q\right)=\frac{1}{N}\langle S_{q}\cdot S_{-q}\rangle$, and the set of $q=k_{F}-k\prime$ that dominates this interfacial response is highlighted in Fig. 4B. Intuitively, the degree of overlap between A(k) of EIO and S(q) of DTO plays a central role in determining the magnitude of interfacial resistance $\rho_{\text{int}}$.

Within this model, we compute $\rho_{\text{int}}$ versus H applied along [111], which reveals an anomalous bump feature that is qualitatively consistent with the experimental observation (Figs. 2A and 4D). Our theoretical modeling predicts smaller values of the magnetic fields at the spin ice–kagome ice crossover and the kagome ice–magnetic monopole transition; this is because the MC simulations were performed in a bulk spin ice system with periodic boundary conditions in three directions, which omit the open boundary effects of the top surface and the demagnetization (shape anisotropy) effects intrinsic to thin-film geometry, both known to quantitatively shift the phase boundaries (*68*–*74*). The calculation asserts that the bump feature develops when the DTO layer enters the kagome ice phase and disappears in the magnetic monopole phase. In particular, the integral kernel, ${|T_{k_{F}k\prime }|}^{2}\left(1-\text{cos}\theta_{k_{F}k\prime }\right)$ (Fig. 4D, inset), demonstrates that the decrease in $\rho_{\text{int}}$ arises from the vanishing of S(q) near the Brillouin zone (BZ) boundary upon increasing magnetic fields.

Further, with a magnetic field H aligned perpendicular to [111], the computed interfacial resistivity, $\rho_{\text{int}}$, accurately yields the experimentally observed sixfold anisotropic behavior and the angular narrowing of MR_ϕ_ (see Fig. 3). As shown in Fig. 4E, the extrema in $\rho_{\text{int}}$ coincide with the q=X and q=0 phases of DTO, achieving maximum and minimum MR_ϕ_ values, respectively. More precisely, because of the negligible lattice mismatch at the interface, $\rho_{\text{int}}$ reaches its maximum as the integral kernel ${|T_{k_{F}k\prime }|}^{2}\left(1-\text{cos}\theta_{k_{F}k\prime }\right)$ contributes most near the BZ boundary (Fig. 4E, top panel insets). Here, the scattering vectors q from the Weyl pockets of EIO strongly “resonate” with DTO’s structure factor S(q), thus preferentially picking up the signal in the q=X phase compared to the q=0 phase. In addition, as magnetic field strength keeps increasing, the q=X phase in DTO appears within a narrow angular range around 90° of ϕ. As such, the resultant $\rho_{\text{int}}$ peaks become narrower at higher magnetic fields, accounting for the experimental angular narrowing effect in MR_ϕ_ (see Fig. 4E).

Overall, our theoretical framework provides a coherent explanation for the observed highly anisotropic transport phenomena in angular-dependent MR, notably the sixfold anisotropic behavior and angular narrowing effect. The observed responses can thus be attributed to the magnetic field–tuned interplay between EIO’s Weyl Fermi arcs, the scattering factor of DTO’s magnetic states, and the interfacial Kondo coupling between the Weyl fermions and Dy spins. We note that our theoretical explanation of the sixfold anisotropy and the angular narrowing effect does not require a full long-range ordered q=X magnetic structure at higher fields. We expect that a quasi–long-range ordered q=X phase would still allow qualitatively consistent results for explaining the experiments but with reduced peak values in the computed $\rho_{\text{int}}$ (see section 1.8.2 of the Supplementary Text for discussions). We also note that, according to (*75*), there is no equilibrium long-range ordered q=X phase under a field-cooled procedure. Finally, the inability of ${\hat{H}}_{\text{int}}$ to capture the twofold symmetry-broken state arising at 9 T implies the nonperturbative and conceivably nonlocal nature of the state, which calls for further exploration (see section 1.16 of the Supplementary Text for discussions on its possible mechanisms).

## DISCUSSION

It is a striking observation that the topological surface states of EIO exhibit the sensitivity required to detect the distinct magnetic phases of DTO. This detection is feasible only when the geometry of the topological Fermi arcs has sufficient overlap with the magnetic structure factor of DTO. Also, our calculations emphasize the crucial importance of the Weyl semimetal phase of EIO for the anisotropic transport behavior at the EIO/DTO interface. Remarkably, due to the intertwined nature of the interfacial coupling, the anisotropic transport response in EIO/DTO provides unique microscopic insights into not only the magnetic properties of DTO but also the topological properties of EIO.

The ability to explore the complex magnetic states of spin ice via the electrical properties in the EIO/DTO heterostructure is central to our findings of the multifaceted interface-driven phenomena, including the magnetic field–induced electronic anisotropy and rotational symmetry breaking. The demonstrated interfacial phenomena within the EIO/DTO pyrochlore heterostructure underpin the strategy that can be extended to seek proximity-induced topology and exotic magnetism across the spectrum of frustrated quantum pyrochlores where conventional probes fail (*22*). For instance, in EIO/DTO, replacing Dy with Tb within the DTO layer transforms it from classical into quantum spin ice (*22*, *76*–*81*), creating an unprecedented synthetic two-dimensional heavy-fermion structure in the presence of emergent dynamical gauge fields.

## MATERIALS AND METHODS

### Sample fabrication

This study uses two types of samples: EIO/DTO and EIO/YSZ. For the EIO/DTO data in the main text as well as figs. S1, S2 (A and B), and S4 to S7, the sample is [111]-oriented EIO(37 nm) on DTO(13 nm) on YSZ; for the EIO/DTO data in fig. S2C, the sample is [111]-oriented EIO(29 nm) on DTO(17 nm) on YSZ; for the EIO/YSZ data, i.e., the control sample results mentioned in the main text as well as in fig. S3, the sample is [111]-oriented EIO(37 nm) on YSZ.

The samples in the study are fabricated by the pulsed laser deposition method. The samples’ proper pyrochlore phases, high crystallinity, and thickness are confirmed by a combination of methods, including in situ reflection high-energy electron diffraction, x-ray reflectivity, x-ray diffraction, and reciprocal space mapping. The nearly perfectly ordered, atomically sharp interface with a chemically ideal arrangement of ions is enabled by the newly developed in situ solid-state epitaxy method [see (*35*) for more details]. We also perform zero-field temperature-dependent resistivity measurements to confirm the existence of a metal-to-insulator–like transition at ≈105 K. The low-temperature magnetotransport measurements are performed after the samples are characterized by all the above methods. Also, other samples synthesized under the same growth conditions are characterized by x-ray photoemission spectroscopy, scanning tunneling electron microscopy, and electron energy loss spectroscopy. Notably, when synthesizing the samples under the same growth conditions, the samples are reproducible in terms of showing consistent properties to the above characterizations. See (*35*) for more details regarding the synthesis and characterizations.

### Electrical transport measurement

The standard 4-probe resistivity method is performed in a top-loading dilution fridge system with a base temperature of ≈20 mK at the National High Magnetic Field Lab, Tallahassee, Florida. The measurements were done using AC current excitations of 1 μA at 5 to 50 Hz using a combination of the current source, CS580, synchronized with lock-in amplifiers, SR830, SR860, or SR865A.

We fabricate bar-like devices with a length of ≈1 to 3 mm and a width of ≈0.3 to 0.5 mm. We ensure a uniform current flow and ohmic contacts. The dimensions used for determining resistivity are measured under optical microscopy, with which we determine an error bar of ≈5% for the absolute resistivity values reported in this study. The samples are attached to a homebuilt 16-pin dip socket by the GE Vanish. The 16-pin socket is put on a homebuilt probe with twisted wires connecting the samples and the electronics. The data are recorded by a homebuilt data acquisition system based on LabView. For all the results, the current is applied along the [1-10] direction.

Specifically, the MR data are measured when the magnetic fields are controlled with a sweeping rate at ≈0.1 T/min; MR is measured for both positive and negative fields, and the results are symmetrized under standard procedures. The angular-dependent MR data are measured when the magnetic fields are fixed at a certain field for over 60 s; angular-dependent MR is measured for both positive and negative fields, and the results are symmetrized under standard procedures. The rotation is achieved by a step motor that controls the tension of the Spring attached to the probe. The angle is calibrated by a Hall sensor that is attached to the probe. The temperature dependence results in figs. S4 and S5 are measured by inputting a DC current on a 50-ohm heater attached near the sample space. Below 1 K, the temperature reading is recorded by the RuOx thermometer near the sample base.

## Supplementary Materials

### This PDF file includes:

Supplementary Text Figs. S1 to S21 References
