# Supplementary material for: Electronic anisotropy and rotational symmetry breaking at a Weyl semimetal/spin ice interface
Source: Sci Adv. 2025 Jun 13;11(24):eadr6202. doi: 10.1126/sciadv.adr6202 (PMC12164986; doi:10.1126/sciadv.adr6202)
Supplement: Supplementary file 1 — Supplementary Text Figs. S1 to S21 References [file sciadv.adr6202_sm.pdf]

Science Advances
AAAS

Supplementary Materials for

Electronic anisotropy and rotational symmetry breaking at a Weyl semimetal/ spin ice interface

Tsung-Chi Wu *et al.*

Corresponding author: Tsung-Chi Wu, tcwu@physics.rutgers.edu; Jak Chakhalian, jak.chakhalian@rutgers.edu

Sci. Adv. **11**, eadr6202 (2025)
DOI: 10.1126/sciadv.adr6202

This PDF file includes:

Supplementary Text
Figs. S1 to S21
References

1 Supplementary Text

1.1 Raw Data of the Main Figures

See Fig. S1 on page 37. Fig. S1 shows the raw data of the main figures.

Fig. S1A shows the raw data of Fig. 3A, B, and C before the data are normalized to ϕ = 0°.

1.2 Data Reproducibility

See Fig. S2 on page 38. Fig. S2 shows the data reproducibility.

Fig. S2A shows the angular-dependent MR of EIO/DTO with the opposite scanning direction (360° to 0°) and four times slower sample rotating speed compared to Fig. S1. It reproduces the features in Fig. 3.

Fig. S2B shows the angular-dependent MR of EIO/DTO Device #2 of Sample #1 (the data in the main text is from Device #1 of Sample #1). It reproduces the features in Device #1 of Sample #1.

Fig. S2C shows angular-dependent MR of EIO/DTO on Sample #2 in Experiment #2. Compared to the data in the main text (Experiment #1), in Experiment #2, we mounted a new sample on a new sample rotator on a different sample stage at room temperature, before putting the sample into the dilution fridge. It reproduces the features in Device #1 of Sample #1.

1.3 Controlled Study on EIO/YSZ

See Fig. S3 on page 39. Fig. S3 shows the results of a controlled study on EIO/YSZ.

Fig. S3A shows the raw data of angular-dependent MR for a fast scan from 360° to 0°.

Fig. S3B shows the raw data of angular-dependent MR for a slow scan from 0° to 360°, where the sample rotating speed is four times slower than that in Fig. S3A. Both scans reveal dramatically different results compared to EIO/DTO.

Fig. S3C shows the longitudinal voltage V_{xx} versus T from 2.5 K to 20 mK, i.e., the raw data of the inset of Fig. 1C.

Fig. S3D shows MR under H // [111] at 20 mK, revealing no bump feature.

Fig. S3E shows the temperature-dependent resistivity from 2 K to 300 K, revealing a similar transition at $\approx$ 105 K (see Fig. 1C).

1.4 Background Subtraction of MR Bump under H // [111]

See Fig. S4 on page 40. Fig. S4 shows the background subtraction of the MR Bump under H // [111] as in Fig. 2A.

First, the background of the data at 20 mK is fitted by a polynomial up to the 4th order from 0 T to 6 T. The bump in the inset is then obtained by subtracting the background from the raw data. On the other hand, the data at 700 mK can be well fitted by a polynomial up to 4th order, leaving no bump feature.

1.5 Temperature Dependence of MR Bump under H // [111]

See Fig. S5 on page 41. Fig. S5 shows the T dependence of the MR bumps under H // [111].

Fig S5A shows MR under H // [111] at T = 20 mK, 140 mK (shifted by $-0.5\,\%$ for visual clarity), 400 mK (shifted by $-1\,\%$), and 700 mK (shifted by $-1.5\,\%$). The inset shows bump amplitudes (see Fig. S4 for how to extract the bump) versus T. T_{SI} is determined to be 700 mK, where no bump is observed.

1.6 Shape Anisotropy and MR Bump under H // [111]

See Fig. S6 on page 42. Fig. S6 shows the shape anisotropy and the MR bump under H // [111].

Fig. S6A shows MR under H // [111] at 20 mK for EIO/DTO, where H_{c2}^{*} and H_{c2}^{**} are defined as the start and the end of the bump, respectively.

Fig. S6B shows the magnetization (M) versus H under H // [111] for bulk DTO, thin-plat DTO, and thin-film DTO. In EIO/DTO, the DTO's M-H curve is shifted to the right due to the shape anisotropy of thin films. H_{c2}^{*} and H_{c2}^{**} correspond to the start and end of the transition from the kagome ice phase to the monopole phase. Bulk DTO and thin-plat DTO results are retrieved from (*27*).

1.7 cos(6ϕ) Fitting of Angular-dependent MR

See Fig. S7 on page 43. Fig. S7 shows the results of $\cos(6\phi)$ Fitting of angular-dependent MR of EIO/DTO (also see Fig. 3).

While angular-dependent MR at $2\,\mathrm{T}$ (**A**) and $3\,\mathrm{T}$ (**B**) can be fit well by $\cos(6\phi)$, angular-dependent MR at $5\,\mathrm{T}$ (**C**) and $18\,\mathrm{T}$ (**D**) cannot.

Fig. S7E shows that the full width at half maximum (FWHM) of the peaks at $\phi = 90°$ is $\approx$ $32°$, $11°$, and $6°$ for $2\,\mathrm{T}$, $9\,\mathrm{T}$, and $18\,\mathrm{T}$, respectively.

Moreover, as shown in Fig. S7F, by analyzing angular MR, which is defined as $\Delta\rho_{xx}(H)/\Delta\phi(H)$ with $\Delta\rho_{xx}(H)$ and $\Delta\phi(H)$ being the amplitudes and FWHM of the peaks at $\phi = 90°$, respectively, we find angular MR of $18\,\mathrm{T}$ increases by $\approx 600\,\%$ compared to that of $2\,\mathrm{T}$.

See section 2.9 for the fitting results obtained using a more systematic approach based on the Bayesian inference model (also shown in Fig. 3C in the main text).

1.8 Theory – Phenomenological Description of the Magnetoresistance of the EIO/DTO Heterostructure

In the following section, we provide a phenomenological theoretical description of the experimental observations of EIO on DTO. The full model of the system is given by $\hat{\mathcal{H}} = \hat{\mathcal{H}}_{\text{EIO}} + \mathcal{H}_{\text{DTO}} + \hat{\mathcal{H}}_{\text{int}}$. Here, $\hat{\mathcal{H}}_{\text{EIO}}$ represents the tight-binding Hamiltonian that describes the slab EIO. Here, in the next few sections, we will explain the details of $\mathcal{H}_{\text{DTO}}$ and $\hat{\mathcal{H}}_{\text{int}}$. The features in the observed magnetoresistance MR_θ and MR_ϕ, including the bump in $\text{MR}(H)$ with $H \parallel [111]$, the MR_θ with a magnetic field tilted away from the [111] direction, and the anisotropic six-fold MR_ϕ when $H \perp [111]$ are explained using a perturbative treatment of $\hat{\mathcal{H}}_{\text{int}}$, the Kondo coupling between the EIO and the DTO at their interface.

1.8.1 Fermi Arc Resistivity Plateau

The longitudinal resistivity results in the main text maintain a constant value (plateau) towards zero temperature and decay as the temperature increases. While the decay of the resistivity can be understood by the linear increase of conductivity with temperature in the gapless Weyl semimetal phase (*82*), the conductivity plateau is induced by the Fermi arc states at the surfaces of the EIO slab.

The EIO slab is treated using a tight-binding model $\hat{\mathcal{H}}_{\text{EIO}}$ derived for the Ir t_{2g} states given by the DFT+U calculations as described in Ref. (*37*). The antiferromagnetic ground state on the pyrochlore lattice features the all-in-all-out magnetic structure and, for certain values of the local Hubbard interaction U (1.05 eV $\leq U_{\text{Ir}} \leq$ 1.45 eV), realizes a topological Weyl semimetal with 24 Weyl nodes. The Fermi arc surface states on the [111] slab produce a metallic density of states at the Fermi energy. Fig. S8(a) shows the total spectral function of an 11-layer EIO slab, while (b) shows the projection onto the triangular and the kagome terminations. It has been shown that the Fermi arc surface states can contribute to large DC conductivity in Weyl

semimetals (*42*). Below, we theoretically show the Fermi arc surface contribution to the DC conductivity by finite slab calculation and identify the resistivity plateau at low temperatures due to surface states.

The conductivity tensor of 2D systems can be expressed as

$$\sigma = \begin{pmatrix} \sigma_{xx} & \sigma_{xy} \\ -\sigma_{xy} & \sigma_{xx} \end{pmatrix}, \tag{1}$$

where x, y directions of the material are considered to be isotropic and infinite (extends in real space to a length scale much larger than that in the z direction). For a strictly linear dispersion around the Weyl points, the diagonal DC conductivity follows the standard Drude model description of the longitudinal conductivity, which depends linearly on the density of states at the Fermi level and the diffusion among the bulk states. In contrast, the off-diagonal part is the anomalous Hall effect, which stems from Berry-curvature singularities of the Weyl points (*83*). The resistivity tensor is the inverse of the conductivity

$$\rho = \frac{1}{\sigma_{xx}^2 + \sigma_{xy}^2} \begin{pmatrix} \sigma_{xx} & -\sigma_{xy} \\ \sigma_{xy} & \sigma_{xx} \end{pmatrix}, \tag{2}$$

with the DC resistivity $\rho_{xx} = \frac{\sigma_{xx}}{\sigma_{xx}^2+\sigma_{xy}^2}$. From the previous experimental results (*37*), the ρ_{xx} is of order $\mathrm{mohm} \cdot \mathrm{cm}$ while the anomalous Hall conductivity σ_{AHE} is of order $ohm^{-1} \cdot \mathrm{cm}^{-1}$, which indicates that $\sigma_{xx} \gg \sigma_{xy}$. Thus, the DC resistivity can be simplified as $\rho_{xx} \approx 1/\sigma_{xx}$.

In our calculations, we chose the tight-binding model derived using DFT+U with $U = 1.4\,\mathrm{eV}$. The numerical calculation for the spectrum and longitudinal conductivity is carried out with the Kernel polynomial method (KPM) implemented on graphical processing units (GPUs) used in previous literature (*37,84–87*). The numerical results of the Fermi arc contribution to the spectrum (the DOS and surface DOS) longitudinal resistivity are shown in Fig. S9. In the closed geometry (periodic/twisted boundary in the z direction), the total DOS $\rho(E) = \frac{1}{N}\sum_{n=1}^{N} \delta(E - E_n)$ vanishes at the Fermi energy, originating from the Weyl semimetallic behavior. In contrast

to the bulk, the slab geometry built with finite number of layers and open boundary conditions in the z direction gives a finite total DOS. When projecting the spectrum to the surface

$$\rho_{\text{sur}}(E) = \frac{1}{A}\sum_{R}\sum_{n} |\langle R|n\rangle|^2 \delta(E - E_n), \tag{3}$$

where R is the position on the surface (top or bottom), and A is the area of the surface.

The longitudinal conductivity and resistivity are presented in Fig. S10. Due to the surface contribution, the resistivity saturates to some finite value at low temperature, which becomes divergent in bulk Weyl semimetals, the closed periodic boundary condition (PBC) geometry. What's more, these saturation values converge with increasing layers (layer $L_z = 31, 61$), manifesting the finite Fermi arc contribution in the resistivity. The computed ρ_{xx}, also plotted in Fig. 4A in the main text, are consistent with the experimental behavior shown in Fig. 1C in the main text.

1.8.2 Monte Carlo Simulation of the Spin Configurations in DTO with Magnetic Field

The DTO is described using the dipolar Hamiltonian H_{DTO}, well-known to describe classical spin ice,

$$\mathcal{H}_{\text{DTO}} = -J\sum_{\langle ij\rangle} \boldsymbol{S}_i^{z_i}\cdot\boldsymbol{S}_j^{z_j} + Dr_{\text{nn}}^3\sum_{j>i}\left[\frac{\boldsymbol{S}_i^{z_i}\cdot\boldsymbol{S}_j^{z_j}}{|\boldsymbol{r}_{ij}|^3} - \frac{3\left(\boldsymbol{S}_i^{z_i}\cdot\boldsymbol{r}_{ij}\right)\left(\boldsymbol{S}_j^{z_j}\cdot\boldsymbol{r}_{ij}\right)}{|\boldsymbol{r}_{ij}|^5}\right], \tag{4}$$

where the Ising moment is normalized to $|\mathbf{S}_i| = 1$, the dipolar interaction strength is $D_{\text{nn}} = \frac{5}{3}D \sim 2.35\text{K}$, and the nearest-neighbor interaction $J_{\text{nn}} = \frac{J}{3}$ is a free parameter of the theory, which is chosen as $J_{\text{nn}} = -1.24\text{K}$ for bulk DTO (*88*). A magnetic field is then added to the system by including an additional term $-\boldsymbol{H}\cdot\sum_i \boldsymbol{S}_i$, where $\boldsymbol{H} = H(\sin\theta\cos\phi, \sin\theta\sin\phi, \cos\theta)$.

The Monte Carlo simulation with Metropolis update was performed, where the dipole-dipole interaction was treated with the standard Ewald summation technique. To conveniently obtain the surface of DTO along [111] direction, we used an enlarged unit cell of the pyrochlore

lattice that contains 12 sublattices located on 3 triangular and 3 kagome layers, such that the layers are perpendicular to the original [111] direction. The simulation was performed on $4 \times 4 \times 1$ (192 sites) and $8 \times 8 \times 2$ (1536 sites) lattices with periodic boundary conditions. For both system sizes, we used 5×10^5 sweeps to anneal the system down to the target temperature starting from 7 K, another 5×10^5 sweeps to further equilibrate the spins, and another 10^6 sweeps for measurements. Typically, we used 4 up to 16 independent initial spin configurations to estimate the statistical errors. As a benchmark, for the zero-field case, our implementation reproduces the sharp peak around 1.5 K in the specific heat of a pyrochlore lattice in Ref. (*88*). With [111] magnetic field, the bulk DTO shows a crossover from spin ice (SI) phase to kagome ice (KI) phase around 0.15 T, and a transition to a magnetic monopole (MM) phase around 0.9 T (see Fig. 4D in the main text). With a tilted magnetic field from [111] direction, DTO shows similar transitions from SI to KI, then to MM, with small θ; for $\theta = 90°$ it only shows a transition to a 2I2O $q = X$ phase. Fig. S11 shows the $S(\mathbf{k} = \mathbf{0})$ with tilted magnetic field along the $\phi = 0°, 20°, 25°, 30°$ direction.

We anticipate that a slight deviation from the fully-developed long-range ordering of the q=X phase will change quantitatively the interfacial resistivity (i.e. renormalizing the peak values) while keeping most of the qualitative features for explaining the six-fold anisotropy. Further studies using MC in larger simulation cells or even open boundary conditions in the kagome surfaces at all 4 π angles are needed to confirm it.

Note that in our Monte Carlo simulation, the Dy moments in DTO are modeled as Ising spins constrained to take either spin up or down values along their local [111] axes, corresponding to the crystal structure's corner-sharing tetrahedra. For convenience in calculations, these local Ising spins are represented as three-dimensional vectors to reflect the orientation of each local Ising axis within the global coordinate system.

For an in-plane magnetic field, DTO shows alternating $q = 0$ (zero momentum) and $q = X$

(finite momentum) phases as the magnetic field rotates every $30°$ (see Fig. 4E upper panel for the spin configurations). Consider the spins in the kagome plane, which can be separated into three sublattices. As the magnetic field rotates by every $120°$, perpendicular to one of the apical spin directions, the spins at the corresponding sublattice get frustrated, while the rest of the spins in the kagome plane are aligned with the field direction. Fig. S13 shows the spin structure factor with a larger in-plane magnetic field.

For each magnetic field, we used 5 × 10^5 sweeps to anneal the system down to the target temperature starting from 7 K, another 5 × 10^5 sweeps to further equilibrate the spins, and another 10^6 sweeps for measurements. Typically, we used 4 up to 16 independent initial spin configurations to estimate the statistical errors. For each of the initial spin configuration, we take 20 snapshots after equilibration. The $S(k)$ of all the spins in a kagome layer is computed as follows. We first identify the three different sublattices in the kagome plane, then for each sublattice labeled by j ($j = 1, 2, 3$), then

$$S(\mathbf{k}) = \frac{1}{N^2} \sum_j \sum_{\mathbf{R}_1, \mathbf{R}_3 \text{ in sublat } j} e^{i\mathbf{k}-(\mathbf{R}_1 - \mathbf{R}_2)} \left\langle \mathbf{S}_{j,\mathbf{R}_1} \cdot \mathbf{S}_{j,\mathbf{R}_2} \right\rangle , \tag{5}$$

where $\mathbf{S}_{j,\mathbf{R}_1}$ is a 3D spin vector at a given site at $\mathbf{R}_1$ from sublattice j. For a 8×8×2 simulation cell, we need to sum over all 64 $\mathbf{R}_1$ vectors. As expected, $S(\mathbf{0})$ is peaked at the zero-momentum phases ($\phi = 0°$, $60°$), while $S(\mathbf{b_2}/2)$ is peaked at the finite-momentum phase ($\phi = 30°$).

At any given angle ϕ, there is a phase transition between the $q = 0$ and $q = X$ phases. The critical field strength varies for different ϕ. This is shown in Fig. S19A-D, where we have plotted the spin structure factor $S(\mathbf{k})$ as given by the Monte Carlo simulations for a $8 \times 8 \times 2$ bulk spin ice at momenta $(0, 0, 0)$ and $X = (0, \frac{1}{2}, 0)$, as a function of the angle ϕ and the field strength H respectively. Based on our MC results, below 0.07 T, it is difficult to identify a transition between the $q = 0$ and $q = X$ phases. The corresponding order parameter could be chosen as $S(X)$. Above 0.07 T, as ϕ increases and approaches $90°$, the critical field for this

transition also increases. We also estimated the phase boundary between the $q = X$ and $q = 0$ phases by locating when $S(X)$ drops below 0.02 from our MC results. A schematic phase diagram is shown below in Fig. S19E. While the phase boundaries can be estimated in this way, the order of the transition cannot be determined because of the limited system size in MC.

In both the experiment and the MC simulation, it is difficult and unlikely to achieve the $q = X$ phase with true long-range order (LRO). In the experiment, when the field angle is slightly deviated from 90 degrees, the system is likely to be stuck in a quasi-LRO phase, as suggested by (*75*). Meanwhile, the MC simulations were performed with temperatures larger than 0.5 K instead of at a very low temperature. Therefore, the experimental difficulty in reaching the true LRO $q = X$ phase is effectively mimicked in our MC results. In other words, for small fields, the MC should also suffer from reaching the LRO states if we use very low temperatures and low fields.

We note that if, instead, a quasi-LRO $q = X$ with similar S(k) is established, we still would expect to obtain qualitatively consistent results, despite having a renormalized heights of the peaks in the calculated ρ_{int}; such a renormalization would only affect the quantitative values but not change the qualitative consistency for explaining the six-fold anisotropy in the experiments. However, if there were merely short-range order in the system, it would be insufficient to explain the angular narrowing effect in angular magnetoresistance at high fields and, therefore, inconsistent with the experiments.

1.8.3 Magnetoresistance Response Given by Perturbative Treatment of the Interfacial Kondo Interaction

For the EIO thin layer grown on the DTO slab, based on the STM results (Fig. 1B in the main text), if the terminating layer of Dy sublattice is a kagome layer, the starting Ir sublattice is a kagome layer. Therefore, we model the interfacial coupling via a Kondo coupling between the classical spins of the Dy (kagome lattice) and quantum spins residing at Ir sites (kagome

lattice). As the e_g bands are much higher in energy and can be ignored in the low energy model Hamiltonian, at each Ir site, three t_{2g} orbitals ($d_{xy},\ d_{yz},\ d_{xz}$) are coupled to the f orbitals from the two nearest-neighbor Dy sites (see Fig. 4C for a schematic of the interfacial spin interaction). We simplify this picture by assuming that the hoppings are the same for all $d-f$ channels. Thus, a microscopic model for the EIO-DTO interaction at the interface is given by

$$\hat{\mathcal{H}}_{\text{int}} = \sum_{\mathbf{R}} \sum_{\langle i,j \rangle} \sum_{\alpha,\beta,\sigma} t \left(\hat{c}^{\dagger}_{i\alpha\mathbf{R}\sigma} \hat{d}_{j\beta\mathbf{R}\sigma} + \text{h.c.} \right), \tag{6}$$

where $i = 1, 2, 3$ represents the three Ir sublattices of the kagome termination, and α and β represent the orbital indices at each site. The summation $\sum_{\mathbf{R}}$ goes over all the lattice vectors $\mathbf{R}$ in real space, and $\langle\,\rangle$ means that we only sum over the nearest-neighbor pairs of Ir sites (i) and Dy sites (j) at the interface. Note that this Hamiltonian implicitly includes a projection from the full EIO Hilbert space to the kagome termination. In k-space, one could write the interfacial hopping alternatively as,

$$\hat{\mathcal{H}}_{\text{int}} = \sum_{\mathbf{k}} \sum_{\langle i,j \rangle} \sum_{\alpha,\beta,\sigma} \left(t e^{i\mathbf{k}\cdot\boldsymbol{\delta}_{ij}} \hat{c}^{\dagger}_{i\alpha\mathbf{k}\sigma} \hat{d}_{j\beta\mathbf{k}\sigma} + \text{h.c.} \right), \tag{7}$$

where $\boldsymbol{\delta}_{ij}$ represents the real-space vector that connects site j (Dy) to site i (Ir) (see Fig. 4C for a visual illustration of $\boldsymbol{\delta}_{ij}$). Note that $\hat{c}^{\dagger}_{i\alpha\mathbf{k}\sigma} = \frac{1}{\sqrt{N}} \sum_{\mathbf{R}} e^{i\mathbf{k}\cdot\mathbf{R}} \hat{c}^{\dagger}_{i\alpha\mathbf{R}\sigma}$.

Recall that the tight-binding Hamiltonian of an EIO slab is given by,

$$\begin{aligned} \hat{\mathcal{H}}_{\text{EIO}} &= \sum_{\mathbf{R}} \sum_{ii'} \sum_{\alpha\beta,\sigma\sigma'} \left(t^{\text{EIO}}(\mathbf{R})_{ii',\alpha\beta,\sigma\sigma'} \hat{c}^{\dagger}_{i\alpha\mathbf{R}\sigma} \hat{c}_{i'\beta\mathbf{R}\sigma'} + \text{h.c.} \right) \\ &= \sum_{\mathbf{k}} \sum_{ii'} \sum_{\alpha\beta,\sigma\sigma'} \left[t^{\text{EIO}}(\mathbf{k})_{ii',\alpha\beta,\sigma\sigma'} \hat{c}^{\dagger}_{i\alpha\mathbf{k}\sigma} \hat{c}_{i'\beta\mathbf{k}\sigma'} + \text{h.c.} \right] \\ &= \sum_{\mathbf{k}} \sum_{n} \epsilon_{n\mathbf{k}} \hat{\psi}^{\dagger}_{n\mathbf{k}} \hat{\psi}_{n\mathbf{k}}, \end{aligned} \tag{8}$$

where $\epsilon_{n\mathbf{k}}$ represents the nth eigenstate at momentum $\mathbf{k}$, and the $\hat{\psi}^{\dagger}_{n\mathbf{k}}$ creates an electron at band n with momentum $\mathbf{k}$. Since the interfacial hopping only acts on the sites at the kagome termination of EIO, we will need to insert a projection operator $\widehat{\mathcal{P}}_{\text{K-term}} = \sum_{\substack{i\alpha\sigma, \\ i\in\text{K-term}}} |i\alpha\sigma\rangle \langle i\alpha\sigma|$,

when rewriting the equations in terms of the EIO slab eigenstates. One can utilize the results of the Schrieffer-Wolff transformation of an Anderson model that is built from this hybridization Hamiltonian and write down directly the Kondo Hamiltonian at the interface,

$$\begin{aligned}\hat{\mathcal{H}}_{\mathrm{K}} &= \frac{1}{\sqrt{N}}\sum_{\mathbf{k},\mathbf{q}}\sum_{\langle i,j\rangle,\alpha,ss'} J_{ij,\mathbf{k}+\mathbf{q},\mathbf{k}}\mathbf{S}_{j\mathbf{q}}\cdot\hat{c}^{\dagger}_{i\alpha\mathbf{k}+\mathbf{q},s}\boldsymbol{\sigma}_{ss'}\hat{c}_{i\alpha\mathbf{k},s'} \\ &= \frac{1}{\sqrt{N}}\sum_{\mathbf{k},\mathbf{q}}\sum_{\langle i,j\rangle,\alpha,ss'} J_{ij,\mathbf{k}+\mathbf{q},\mathbf{k}}\mathbf{S}_{j\mathbf{q}}\cdot\hat{\psi}^{\dagger}_{\mathbf{k}+\mathbf{q}}\langle\mathbf{k}+\mathbf{q}|i\alpha s\rangle\,\boldsymbol{\sigma}_{ss'}\hat{\psi}_{\mathbf{k}}\langle i\alpha s'|\mathbf{k}\rangle\,.\end{aligned} \tag{9}$$

where $\mathbf{S}_i$ is a classical O(3) vector characterizing the local dipole moments at the Dy sites in DTO. We have simplified the problem by considering only the closest band near the Fermi level; therefore, the band index for operator $\hat{\psi}^{\dagger}_{\mathbf{k}}$ is omitted. For the coupling channel between Ir site i and Dy site j, the Kondo coupling $J_{\mathbf{k}+\mathbf{q},\mathbf{k}}$ is given by,

$$J_{ij,\mathbf{k}+\mathbf{q},\mathbf{k}} \propto \frac{t_{\mathbf{k}+\mathbf{q}}t^{*}_{\mathbf{k}}}{U} = \frac{|t|^2}{U}e^{i\mathbf{q}\cdot\boldsymbol{\delta}_{ij}}, \tag{10}$$

where U is the on-site, intra-orbital Hubbard interaction strength for the Dy f electrons.

For an 11-layer EIO slab, we have identified the closest eigenstate near the Fermi level to couple to the Dy spins, while all the other eigenstates lie at least 5 meV away from the Fermi level. As the biggest spacing between layers is at the interface in conjunction with the non-compatibility of the atomic orbitals in the two materials, it leads us to argue that the Kondo coupling is the smallest interaction scale in the problem and can be treated perturbatively. By solving the Boltzmann equation, we get the expression for the conductivity at the interface due to the presence of the lattice Kondo interaction above:

$$\sigma_{\text{int}} = \frac{e^2}{3}\sum_{n}\int \mathrm{d}\mathbf{k}\tau(n,\mathbf{k})|\mathbf{v}_{n\mathbf{k}}\cdot\hat{\mathbf{j}}|^2\left(-\frac{df_0(n,\mathbf{k})}{d\epsilon_{n\mathbf{k}}}\right), \tag{11}$$

where $\mathbf{v}_{n\mathbf{k}} = \nabla_{\mathbf{k}}\epsilon_{n\mathbf{k}}$, and $\hat{\mathbf{j}}$ is the unit vector along the current. $f_0(n,\mathbf{k})$ is the filling at equilibrium of band n with momentum $\mathbf{k}$ (we have reintroduced the index n, which differs for different k, to represent the band index, and suppressed the sublattices i and orbitals α into n). Assuming

elastic scattering, the relaxation time $\tau(n,\mathbf{k})$ of mode n and momentum $\mathbf{k}$ is given by (*89*)

$$\tau^{-1}(n,\mathbf{k}) = \sum_{ss'} \int \mathrm{d}\mathbf{k}' \delta(\epsilon_{n\mathbf{k}'} - \epsilon_{n\mathbf{k}}) |T_{\mathbf{k}\mathbf{k}',ss'}|^2 \left(1 - \cos\theta_{\mathbf{k}\mathbf{k}'}\right). \tag{12}$$

From Eq. 9, we know that the tunneling matrix element is given by

$$T_{\mathbf{k}\mathbf{k}',ss'} = \frac{1}{N} \sum_{\langle i,j \rangle} \sum_{\alpha} \frac{|t|^2}{U} e^{i(\mathbf{k}'-\mathbf{k})\cdot\boldsymbol{\delta}_{ij}} \langle \mathbf{k} | i\alpha s \rangle \, \mathbf{S}_{j,\mathbf{k}'-\mathbf{k}} \cdot \boldsymbol{\sigma}_{ss'} \langle i\alpha s' | \mathbf{k}' \rangle \tag{13}$$

where i (j) runs over the three sublattices in EIO (DTO) kagome surfaces. Therefore, we get

$$\begin{aligned}
|T_{\mathbf{k}\mathbf{k}'}|^2 &= \frac{1}{N} \sum_{\langle i,j \rangle,\alpha} \sum_{ss'} |T_{\mathbf{k}\mathbf{k}',ss'}|^2 \\
&= \frac{1}{N} \frac{|t|^4}{U^2} \sum_{\langle i,j \rangle,\alpha} \sum_{ss'} \sum_{ab} S^a_{j,\mathbf{k}'-\mathbf{k}} S^b_{j,\mathbf{k}-\mathbf{k}'} \sigma^a_{ss'} \sigma^b_{s's} | \langle \mathbf{k} | i\alpha s \rangle \langle i\alpha s' | \mathbf{k}' \rangle |^2 \\
&= \frac{1}{N} \frac{|t|^4}{U^2} \sum_{\langle i,j \rangle,\alpha} \sum_{ss'} \sum_{ab} S^a_{j,\mathbf{k}'-\mathbf{k}} S^b_{j,\mathbf{k}-\mathbf{k}'} \delta_{ab} \sigma_{ss'} | \langle \mathbf{k} | i\alpha s \rangle \langle i\alpha s' | \mathbf{k}' \rangle |^2 \\
&= \frac{1}{N} \frac{|t|^4}{U^2} \sum_{\langle i,j \rangle,\alpha} \mathbf{S}_{j,\mathbf{k}'-\mathbf{k}} \cdot \mathbf{S}_{j,\mathbf{k}-\mathbf{k}'} \sum_{s} | \langle i\alpha s | \mathbf{k} \rangle \langle \mathbf{k}' | i\alpha s \rangle |^2,
\end{aligned} \tag{14}$$

where $a,b = x,y,z$. One can think of $\langle i\alpha s|\mathbf{k}\rangle \langle \mathbf{k}'|i\alpha s\rangle$ as the diagonal elements of the one-particle density matrix $|\mathbf{k}\rangle \langle \mathbf{k}'|$ in the localized basis $|i\alpha s\rangle$. Note that one can define the spin structure factor $S(\mathbf{q}) = \frac{1}{N} \langle \mathbf{S}_\mathbf{q} \cdot \mathbf{S}_{-\mathbf{q}} \rangle$. Here, the braket means tracing over the thermal density matrix $e^{-\beta H_{\mathrm{DTO}}}$, which, in practice, corresponds to taking the average value over all the Monte Carlo snapshots. Therefore,

$$|T_{\mathbf{k}\mathbf{k}'}|^2 = |J|^2 \sum_{\langle i,j \rangle} S_j(\mathbf{k}-\mathbf{k}') \sum_{s} | \langle i\alpha s | \mathbf{k} \rangle \langle \mathbf{k}' | i\alpha s \rangle |^2. \tag{15}$$

In order to perform the integral Eq. 12, we need to first identify the $\mathbf{k}_\mathrm{F}$ as given by EIO's electronic structure, then perform the summation over all $\mathbf{k}_\mathrm{F} - \mathbf{k}'$, where $\mathbf{k}' \in \{\mathbf{k}_\mathrm{F}\}$. The computational cost for performing this summation for $\tau^{-1}(\mathbf{k}_\mathrm{F})$ scales with $N^3_{\mathbf{k}_\mathrm{F}}$, and in order to get σ_{int}, another summation over $\mathbf{k}_\mathrm{F}$ is needed, giving rise to a cost of $N_{\mathbf{k}_\mathrm{F}}$. Therefore, to

simplify the procedures, we identify the $\mathbf{k}_\mathrm{F}$ by keeping only the momenta where the projection of the state at the Fermi level onto the kagome termination above a certain value chosen to ensure that the resultant number of $\mathbf{k}_\mathrm{F}$ is about 20, then we perform the summation only on these $\mathbf{k}_\mathrm{F}$'s (indicated by the grey dots in Fig. 4B, left panel)

In EIO/DTO heterostructure: spin ice coupled to a Weyl semimetal with topological surface states Fermi surface structure We computed the resultant interfacial resistivity $\rho_\mathrm{int} = \sigma_\mathrm{int}^{-1}$ given by the input of four different EIO models, derived from DFT+U with U =1.1, 1.2, 1.3, 1.4 eV (in which EIO is a Weyl semimetal), shown in Fig. S14. In the main text, results with $U =$ 1.3 eV are shown in Fig. 4E. The upper panels of Fig. S14(a)-(d) show the projected spectral function on to the kagome termination, with the grey dots representing the identified $\mathbf{k}_\mathrm{F}$ on a uniform 24 $\times$ 24 k sampling of the BZ. We see that the response ρ_int is distinct for different values of U. For $U \leq$ 1.3 eV, the maxima occur near the finite momentum phase (ϕ = 90°), while for $U = 1.4$ eV, the maxima occur at the zero momentum phase (ϕ = 60°, 120°). This can be understood by noting that the Fermi surface profile on the kagome termination depends highly on U, i.e., the locations of the Weyl points. Therefore, the scattering vectors $\mathbf{q} = \mathbf{k}_\mathrm{F} - \mathbf{k}'$, where $\mathbf{k}' \in \{\mathbf{k}_\mathrm{F}\}$ also change with U, picking up the signal of DTO's $S(\mathbf{k})$ from different regions of the BZ (see Fig. S13 lower panels). Curiously, we obtained the two-peak feature near the finite momentum phase in ρ_int that occurs with smaller U, with a much smaller overall value than that given by $U \geq$ 1.3 eV. Further calculations involving a full summation of the BZ are required to resolve whether this is due to the finite size effect in DTO spin configurations, or the fact that we have manually thrown out many of the k points and near-Fermi-level states that also contribute. Therefore, by changing the Fermi surface profile of the metal/semimetal that the spin ice many-body wave function is coupled to, one could end up with various patterns in the ρ_int, manifested as the MR_ϕ pattern measured in the EIO/DTO heterostructure.

Simple case: spin ice coupled to a single-band, normal metal with isotropic Fermi surface around Γ If one were to couple the spins in DTO to a trivial isotropic system with $\mathbf{k}_{\rm F}$ circled at Γ (see Fig. S15A), i.e., a simple single-band, metallic system, using Eq. 12, one would expect it to pick up the maximum value at zero momentum phases. This is because when the density matrix diagonal elements $\sum_s |\langle i\alpha s|\mathbf{k}\rangle \langle \mathbf{k}'|i\alpha s\rangle|^2$ in Eq. 14 are reduced to constants for all the sublattice i and independent of $(\mathbf{k}-\mathbf{k}')$, Eq. 12 can be simplified to

$$\tau^{-1}(\mathbf{k}) \propto \sum_{j=1,2,3} \int \mathrm{d}\mathbf{k}' \delta(\epsilon_{n\mathbf{k}'} - \epsilon_{n\mathbf{k}}) S_j(\mathbf{k}-\mathbf{k}')(1-\cos\theta_{\mathbf{k}\mathbf{k}'}). \tag{16}$$

Since the factor $(1-\cos\theta_{\mathbf{k}\mathbf{k}'})$ is maximized when $\mathbf{k}$ and $\mathbf{k}'$ are in opposite directions, and for this trivial case, both $\mathbf{k}$ and $\mathbf{k}'$ come from a small circle centered around Γ — the computed $\tau^{-1}(\mathbf{k}_{\rm F})$'s behavior is qualitatively the same with $S(\mathbf{k}_{\rm F}) \approx S(\mathbf{k}=0)$. Since DTO's $S(\mathbf{k}=0)$ becomes finite at its 2I2O zero-momentum phase, and vanishes at the finite-momentum phase, the resultant resistivity's maxima would also occur at the spin-ice zero-momentum phase, and the minima at the finite-momentum phase. In particular, it would not show increasingly sharp spikes at higher magnetic field strengths. Fig. S15B shows the results of $\rho_{\rm int}$ in this case.

1.9 A Bayesian Inference Study of the Emergence of Two-Fold Anisotropy in Angular-dependent MR

To better understand the six-fold and the emergent two-fold anisotropy, we fit the measured MR with an in-plane magnetic field to the following model,

$$\begin{aligned}\left[\frac{\Delta\rho_{xx}(\phi, H)}{\rho_{xx}(0^\circ, H)}\right]_{\text{model}} &= \text{MR}_6(\phi, H) + \text{MR}_2(\phi, H) + \text{MR}_0 \\ &= A\frac{1}{\left[\cos^2\left(6\left(\phi + \varphi_1\right)\right) - 1\right]^2/\sigma^2 + 1} + B\cos\left[2\left(\phi + \varphi_2\right)\right] + C.\end{aligned} \tag{17}$$

Here, we have decomposed the signal of MR_ϕ into three parts: apart from the constant shift C, there is a six-fold anisotropic contribution $\text{MR}_6(\phi, H)$, which is modeled using a Lorentzian with width σ and amplitude A, and a two-fold anisotropic contribution $\text{MR}_2(\phi, H)$, which is modeled using a simple cosine function with phase φ_2. We then perform a conventional non-linear least square fit of measured MR vs ϕ, for all field strengths $H \geq 1\,\text{T}$. A summary of the results is plotted in Fig. S16 (scan 1), and in Fig. S17 (scan 2).

Fig. S16A shows the measured $\text{MR}(\phi, H)$ given by the transport measurement of the EIO/DTO sample with an in-plane magnetic field. The y values are shifted for clarity (with zeros indicated by the dashed horizontal lines). The black curves are the results predicted by a fitted model as given by Eq. 17. We can see that the fitted results agree well with the measured MR. Fig. S16B and C show the six-fold and two-fold anisotropic components $\text{MR}_6(\phi, H)$ and $\text{MR}_2(\phi, H)$ vs the field strength H. One can see that $\text{MR}_6(\phi, H)$ narrows as the field strength increases, and the two-fold anisotropy begins to emerge at high fields. Fig. S16D-I show the fitted parameters six-fold amplitude A (also shown in Fig. 3C in the main text, labeled as $|\text{MR}_6|$), two-fold amplitude B (also shown in Fig. 3C in the main text, labeled as $|\text{MR}_2|$), six-fold peaks width σ, phases φ_1 and φ_2 (also shown in Fig. 3C in the main text), and constant C. We can see from panel E that the two-fold anisotropy is always nonzero as predicted by our naïve fit to the model (Eq. 17). Similar conclusions can be drawn from the fitting of the data set obtained with scan 2.

However, the analysis above could be biased. For example, for field strength 2 T, including the two-fold anisotropic part MR_2 in the fitting could lead to over-fitting. The experimentally measured MR_ϕ always has a finite error ϵ (deviation from the predicted value given by the fitted model 17), and one should take this error into account when trying to predict the uncertainty of the two-fold anisotropy amplitude B for the component $\mathrm{MR}_2(\phi, H)$. One way of systematically predicting this value is to perform a Bayesian inference of the probability distribution of B and the measurement error ϵ. When ϵ overshoots B, we conclude that the signal of two-fold anisotropy is so weak and thus can not be resolved in our transport measurement.

Using Bayes theorem, given the measured data, the probability distribution of B and ϵ is given by

$$P(B, \epsilon \mid \text{data}) = \frac{P(\text{data} \mid B, \epsilon) P(B, \epsilon)}{\iint P(\text{data} \mid B, \epsilon) P(B, \epsilon) \mathrm{d}B \mathrm{d}\epsilon}. \quad (18)$$

We assume weakly informed priors. For the two-fold anisotropy amplitude B, we assume that $B \sim U(0,\, 2B_0)$, i.e., B is uniformly distributed between $-4B_0$ and $4B_0$, where B_0 is obtained from a simple non-linear least square fit of the data. For the measurement error ϵ, since it is always positive definite, we assume that it is given by a half-norm distribution of width 10^{-2} (estimated using the raw data). We then use the Python package pymc3 (*90*) to build the Bayesian inference model (Eq. 18), then use Monte Carlo to draw the posterior $P(B,\, \epsilon \mid \text{data})$.

Fig. S18A and D shows the probability distribution of the two-fold anisotropy amplitude B and the measurement error ϵ obtained for scan 1 and 2. At low field strengths, the mean of B is below ϵ, suggesting that the noise in the measurement could be the reason why the naïve least square fit yields a finite B value (Fig. S16E and Fig. S17E). As the magnetic field strength increases, the mean of B overshoots the ϵ, suggesting that now the two-fold anisotropy emerges. This can be seen more clearly in Fig. S18B and E, which shows the ratio $\bar{B}/\bar{\epsilon}$ versus the field strength. When $\bar{B}/\bar{\epsilon} > 1$, the two-fold anisotropy emerges from the measured MR_ϕ, corresponding to a critical field about 7∼9 T, qualitatively consistent with the critical field strength

determined by the ovality. Note that at 1 T, scan 1 shows a clear two-fold anisotropy ($\bar{B}/\bar{\epsilon} > 1$), while scan 2 does not, i.e., the two-fold anisotropy is not reproducible among the two data sets we have. Therefore, in Fig. 3C of the main text, we greyed out the data points corresponding to the two-fold anisotropy at 1 T.

Consistency with the "ovality" quantity that characterizes the two-fold anisotropy of MR

To quantify the two-fold response, we computed the "ovality" quantity versus H. Here we define ovality as $(\rho_a - \rho_b)$, where $\rho_a = [\rho_{xx}(180°) + \rho_{xx}(360°)]/2$, and $\rho_b = [\rho_{xx}(60°) + \rho_{xx}(120°) + \rho_{xx}(240°) + \rho_{xx}(300°)]/4$. As shown in Fig. S18C and F, ovality is non-zero within errorbars (arising from the standard deviation of ρ_a and ρ_b, when averaging over a finite interval) for low fields $H = 1$ T, and at higher fields $H \geq 5$ T. The results are qualitatively consistent with the results given by Bayesian analysis, which indicates that the two-fold anisotropy is very likely to be present for $H = 1$ T and $H \geq 9$ T (Fig. S18A, B, D, E). The two-fold anisotropy signal between 1 T and 9 T is likely to be small and, therefore, is washed out by the noise in the experimental data.

1.10 Limitations in the Theoretical Modeling

We note that our simulations omit the fact that the DTO layer has an open top surface in the Monte Carlo simulation. However, we highlight that the six-fold anisotropy in the magnetoresistance of the heterostructure is directly tied to transitions between finite- and zero-momentum phases under an external magnetic field, which, to our understanding, is likely to remain present in a thin-film geometry, though its enhanced geometric frustrations lead to larger critical magnetic field strengths.

Within our approximation, the top EIO layer does not significantly change the ground state in DTO due to the slow dynamics of its large local spin magnetic moments. Therefore, we started with a minimal model that describes the bulk spin ice, and coupled its Monte Carlo snapshots with the topological surface state from ETO using a phenomenological Kondo lattice model, which led to a qualitatively reasonable explanation of the experimental observations. A more realistic approach would involve a detailed Monte Carlo simulation of the [111] thin-film spin ice under an external magnetic field, which will also capture the demagnetization effect. For EIO, a more comprehensive treatment would include the modulations of the spin textures in the topological surface states after considering the local magnetic fields from the finite spin moments of the DTO top surface. Additionally, going beyond the perturbative approach and performing a full self-consistent treatment of the coupling between the conduction electrons and local moments at the interface presents a challenging yet promising future direction. Finally, ab-initio modeling of the interface is an important next step for achieving a quantitative understanding.

1.11 Comparison between Our Work and Recent Works in Pyrochlore Heterostructures

We comment on the similarities and differences between these works and ours:

In NPJ Quantum Materials 2, 64 (2017) (*25*), the theoretical work outlines a path to topological superconductivity by designing a pyrochlore heterostructure consisting of a quantum spin ice and a metal. In our case, our heterostructure contains neither a quantum spin ice nor a conventional metal; in addition, our work is not connected to topological superconductivity.

In Nat. Comm. 11, 1341 (2020) (*26*), the authors present a theoretical approach to create a two-dimensional magnetic monopole gas using an $A_2Ir_2O_7/A_2Ti_2O_7$ (A = Ho, Dy) pyrochlore heterostructure. This approach requires the same A-site ion in both the titanate and iridate layers and does not rely on the topological properties of $A_2Ir_2O_7$. In contrast, our heterostructure employs different A-site ions and, crucially, $Eu_2Ir_2O_7$ is a magnetic Weyl semimetal that hosts Fermi-arc topological states at the interface.

In Science Advances 13, 6308 (2024) (*24*), the experimental work focuses on detecting spin ice' magnetism via a nonmagnetic and topologically trivial metal. In contrast, our work investigates the emergent interfacial phenomena and states arising from the interaction between spin ice and topological states of the magnetic semimetal. As for Nat. Commun. 14, 1404 (2023) (*27*), it is similarly focuses on detecting spin ice' magnetism with a nonmagnetic, non-topological metal.

1.12 Comments on the Layer Alternance and Oxygen Sublattice Near the Interface

For a pyrochlore lattice with the chemical formula $A_2B_2O_7$ viewed along the [111] direction, each kagome (K) layer of A ions is entwined with a triangular (T) layer of B ions, and vice versa. In Fig. 1C of the main text, we highlight the magnetically active A (Dy) and B (Ir) sublattices. To underscore their magnetic roles, we did not superimpose the magnetically "silent" A (Eu) and B (Ti) sublattices in the STEM-HAADF image.

Moreover, in the STEM-HAADF image, the oxygen sublattice has an extremely low contrast and is therefore difficult to distinguish. Thus, only the heavier cation sublattices are readily identifiable in the images. In (33), we have reported STEM-HAADF, STEM-ABF, and STEM-EELS results; together, these results enable us to identify all ions (including oxygens) by experiments. Details of these STEM results can be found in (33).

1.13 Comments on the Quasi-plateau Feature near 1 K in Fig. 1C

It seems that there is a quasi-plateau feature around 1 K in Fig. 1C. We provide a zoomed-in view of Fig. 1C at low temperatures in Fig. S20. As seen in the right panel, the previously noted quasi-plateau feature is not observed. This implies that the seemingly existing quasi-plateau results from the log-log scale, which amplifies minor fluctuations due to the sample-stage vibration during cooling to sub-Kelvin temperatures.

1.14 Evidence for Interfacial Scattering in EIO/DTO at Zero Magnetic Field

Fig. S21 compares the temperature-dependent resistivity of EIO/DTO and EIO. After normalizing both datasets in the same way (i.e., dividing by the resistivity at 20 mK), distinct differences are found. Because the resistivity in EIO/DTO arises from both contributions within the EIO bulk and the EIO/DTO interface, these differences highlight the effect of interfacial scattering, providing further evidence for the existence of interfacial scattering at zero magnetic field.

1.15 Exclude the Possibility that the Symmetry-broken Phase is Attributed to Bulk $Dy_2Ti_2O_7$'s Magnetization

To demonstrate that the symmetry-broken phase cannot be attributed to the magnetization of bulk $Dy_2Ti_2O_7$ (DTO), we refer to earlier studies on angular-resolved magnetic measurements of bulk DTO (*60–62*). In (*60*), the magnetic susceptibility was measured under a rotating field using a vector magnet at sub-Kelvin temperatures on bulk DTO crystals of approximately 1 mm × 1 mm × 1 mm. The magnetization measured at 0° and 180° shows the same absolute value. Furthermore, by performing measurements at every 60°, the study demonstrated that the bulk magnetization displays a symmetric response, consistent with the ordering of the $q = 0$ phase at every 60° interval within the [111] plane. Thus, the results suggest no evidence for a two-fold bulk magnetization. On the other hand, in (*61, 62*), angular-resolved DC magnetization revealed direct evidence for several magnetic phase transitions of bulk DTO, including the one observed in (61), providing the thermodynamic evidence to corroborate the results of (*60*). Lastly, we note that the experiments from (*60–62*) were performed under magnetic fields no higher than 4 T, lower than the onset of our observed symmetry breaking in EIO/DTO at 9 T.

1.16 Possible Mechanisms for the Rotational-symmetry-broken State at High Magnetic Fields

Here, we discuss several possible mechanisms for the rotational-symmetry-broken state at high magnetic fields.

One potential mechanism is the interplay between the Ruderman-Kittel-Kasuya-Yosida (RKKY) interaction and the dipolar interaction at the interface of EIO/DTO, both of which are long-range interactions. It has recently been found that the interplay between the RKKY interaction, nearest-neighbor exchange interaction, and dipolar interaction is important for explaining the complex ground-state properties of Tsai-type quasicrystals (*91*). To the best of our knowledge, the interplay of these interactions at a Weyl semimetal/spin ice interface have not been thoroughly studied; thus, our work could motivate such studies for the crystalline materials interface systems beyond quasicrystals.

Additionally, it has been found that thin films of spin ice materials can display distinct properties not found in their bulk counterparts (*68–73*). However, we note that the DTO layer of the EIO/DTO heterostructures in this study ranged from 13-17 nm, far from an ultra-thin limit. Additionally, our theoretical model of the interface, based on bulk spin ice's magnetization, successfully captures most of the experimental results except for the high field measurements. Thus, our DTO layer's magnetic properties appear bulk-like, preserving a six-fold but not two-fold magnetization within the (111)-plane [see Sec. 2.15 where we discuss the bulk magnetization results of DTO in detail]. This prevents us from attributing the onset of symmetry breaking exclusively to the DTO layer. Nevertheless, it will still be interesting to investigate theoretically and experimentally: (a) the symmetry-broken phase's dependence on the DTO thickness, especially at the ultra-thin limit; (b) whether the symmetry-broken phase is related to unique behaviors of thin-film spin ice at high magnetic fields; (c) the angular-resolved magnetic properties of bulk DTO in sub-Kelvin temperatures and under high magnetic fields up to 18 T.

It is also tempting to attribute the symmetry breaking to the EIO overlayer. However, our high-resolution scanning tunneling microscopy results have revealed that the EIO overlayer has preserved the pyrochlore symmetry, thus excluding the possibility that the observed two-fold response is solely due to EIO [see Sec. 2.17 for more details]. Moreover, the phase shift between EIO/DTO and the control sample EIO [see Fig. 3] strongly suggests that the observed anisotropy in EIO/DTO bears a new origin.

1.17 Exclude the Origin of Symmetry Breaking from the EIO Overlayer in EIO/DTO

For a pyrochlore lattice with the chemical formula $A_2B_2O_7$ viewed along the [111] direction, each kagome (K) layer of A ions is entwined with a triangular (T) layer of B ions, and vice versa. In Fig. 1C of the main text, we highlight the magnetically active A (Dy) and B (Ir) sublattices. To underscore their magnetic roles, we did not superimpose the magnetically "silent" A (Eu) and B (Ti) sublattices in the STEM-HAADF image.

Moreover, in the STEM-HAADF image, the oxygen sublattice has an extremely low contrast and is therefore difficult to distinguish. Thus, only the heavier cation sublattices are readily identifiable in the images. In (*35*), we have reported STEM-HAADF, STEM-ABF, and STEM-EELS results; together, these results enable us to identify all ions (including oxygens) by experiments. Specifically, the ions in the EIO layer align precisely with the expected atomic positions of $Eu_2Ir_2O_7$ with a conventional pyrochlore structure. The results demonstrate the absence of any symmetry breaking or structural reconstruction, including those potentially arising from defects. Additional details of our STEM/EELS analysis at the EIO/DTO interface can be found in our recent publication (*35*).

1.18 Level of Control of the Interface Quality in EIO/DTO

For a pyrochlore lattice with the chemical formula $A_2B_2O_7$ viewed along the [111] direction, each kagome (K) layer of A ions is entwined with a triangular (T) layer of B ions, and vice versa. In Fig. 1C of the main text, we highlight the magnetically active A (Dy) and B (Ir) sublattices. To underscore their magnetic roles, we did not superimpose the magnetically "silent" A (Eu) and B (Ti) sublattices in the STEM-HAADF image.

Moreover, in the STEM-HAADF image, the oxygen sublattice has an extremely low contrast and is therefore difficult to distinguish. Thus, only the heavier cation sublattices are readily identifiable in the i mages. Moreover, in the STEM-HAADF image, the oxygen sublattice has an extremely low contrast and is therefore difficult to distinguish. Thus, only the heavier cation sublattices are readily identifiable in the i mages. In (33), we have reported STEM-HAADF, STEM-ABF, and STEM-EELS results; together, these results enable us to identify all ions (including oxygens) by experiments. Details of these STEM results can be found in (33). Specifically, the ions in the EIO layer align precisely with the expected atomic positions of $Eu_2Ir_2O_7$ with a conventional pyrochlore structure. We observe no contrast attributable to oxygen vacancies or additional superstructures, thereby ruling out interface reconstruction. For comparison, the STEM data for STO/Al_2O_3 in (*92*), where reconstruction is evident, differ markedly with EIO/DTO.

2 Figures and Tables

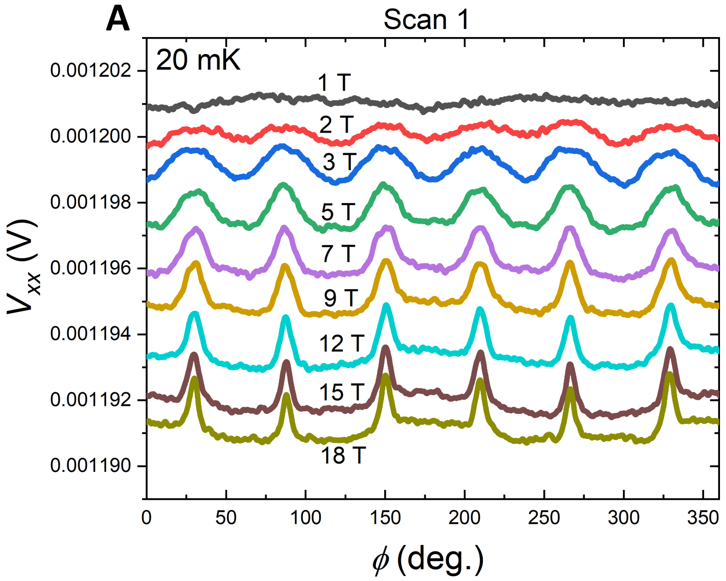

Fig. S1: **Raw Data of Main Figures.** (**A**) Raw Data of Fig. 3A, B, and C (Scan 1)'s results before normalized to $\phi = 0°$.

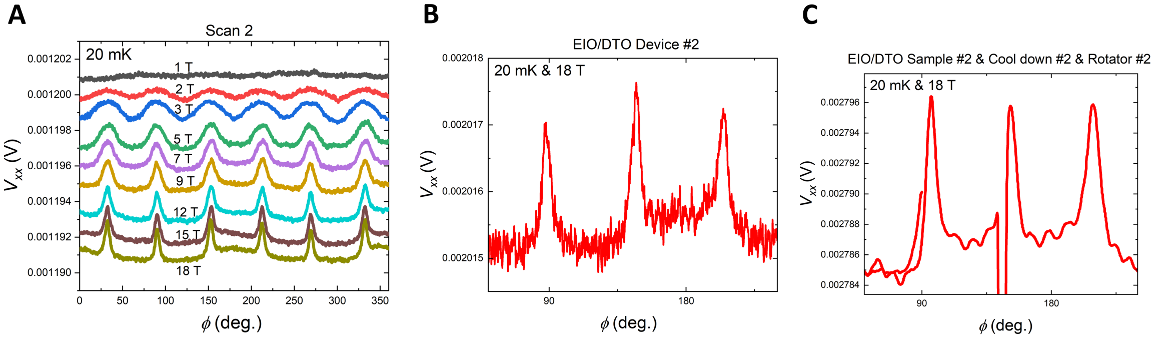

Fig. S2: **Data Reproducibility.** (**A**) Raw angular-dependent MR of EIO/DTO with the opposite and slower scan at 20 mK. The sample rotational speed is four times slower than Scan 1's results [see fig. S1]. (**B**) Raw angular-dependent MR of EIO/DTO Device #2. (**C**) Raw angular-dependent MR of EIO/DTO on Sample #2 in Experiment #2. Compared to the data (Experiment #1) in the main text, in Experiment #2, we mounted a new sample on a new sample rotator on a different sample stage at room temperature before putting the sample into the dilution fridge.

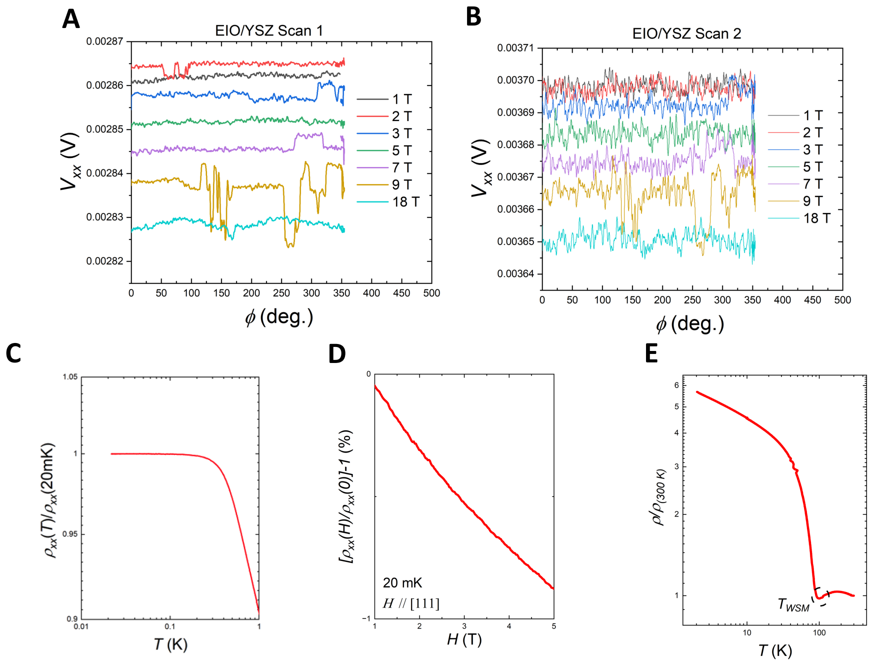

Fig. S3: **Controlled Study on EIO/YSZ.** (**A**) Raw angular-dependent MR data for a quick scan from 360° to 0°. (**B**) Raw angular-dependent MR data for a slow scan from 0° to 360°. (**C**) Longitudinal voltage versus T from 2.5 K to 20 mK. (**D**) MR under H // [111] at 20 mK. (**E**) Temperature-dependent resistivity from 2 K to 300 K.

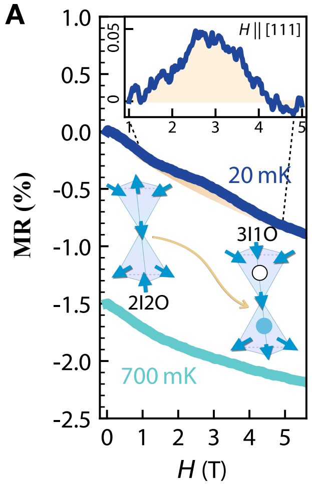

Fig. S4: **Background Subtraction of MR Bump under H // [111].** (**A**) The data background at 20 mK is fitted by a polynomial up to 4th order from 0 T to 6 T. The bump in the inset is then obtained by subtracting the background from the raw data.

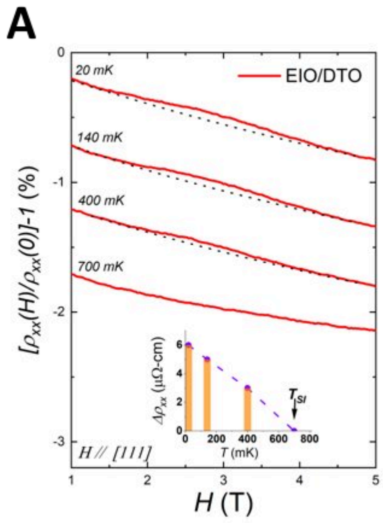

Fig. S5: **Temperature Dependence of MR Bump under** H **// [111].** (**A**) MR under H // [111] at T = 20 mK, 140 mK (shifted for -0.5 %), 400 mK (shifted for -1 %), and 700 mK (shifted for -1.5 %) are shown. The inset shows bump amplitudes (see fig. S4) versus T. T_{SI} is defined at 700 mK, where no bump is observed.

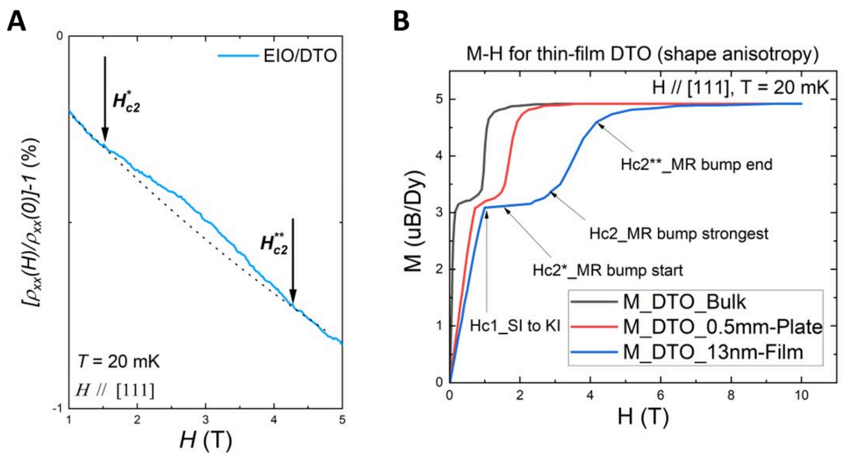

Fig. S6: **Shape Anisotropy and MR Bump under H // [111].** (**A**) MR under H // [111] at 20 mK for EIO/DTO, where H_{c2}^{*} and H_{c2}^{**} are defined as the start and the end of the bump, respectively. (**B**) Magnetization (M) versus H under H // [111] for bulk DTO, thin-plat DTO, and thin-film DTO are shown. In EIO/DTO, the DTO's M-H curve is shifted to the right due to the shape anisotropy of thin films. H_{c2}^{*} and H_{c2}^{**} correspond to the start and end of the transition from the kagome ice phase to the monopole phase.

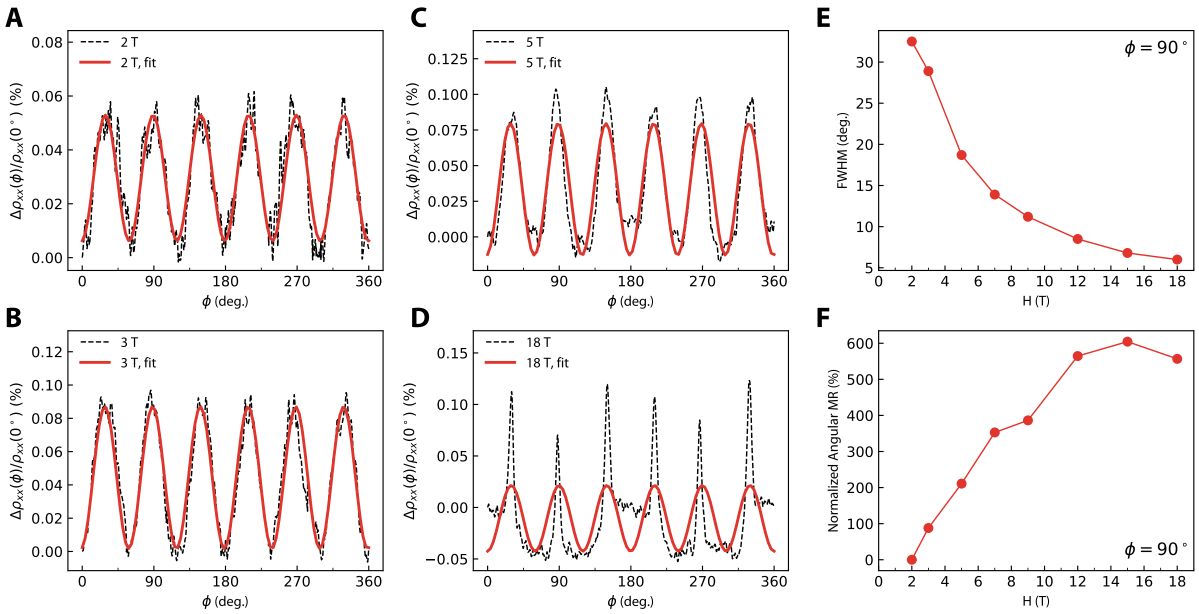

Fig. S7: **Cos6ϕ Fitting of angular-dependent MR.** While angular-dependent MR at 2 T (**A**) and 3 T (**B**) can be fit well by Cos6ϕ, angular-dependent MR at 5 T (**C**) and 18 T (**D**) cannot. (**E**) shows that the full width at half maximum (FWHM) of the peaks at $\phi = 90^\circ$ is $\approx 32^\circ$, 11°, and 6.0° for 2 T, 9 T, and 18 T, respectively. (**F**) shows angular MR, which is defined as $\Delta\rho_{xx}(H)/\Delta\phi(H)$ with $\Delta\rho_{xx}(H)$ and $\Delta\phi(H)$ being the amplitudes and FWHM of the peaks at $\phi = 90^\circ$, respectively. Angular MR of 18 T increases by $\approx 600\,\%$ compared to that of 2 T.

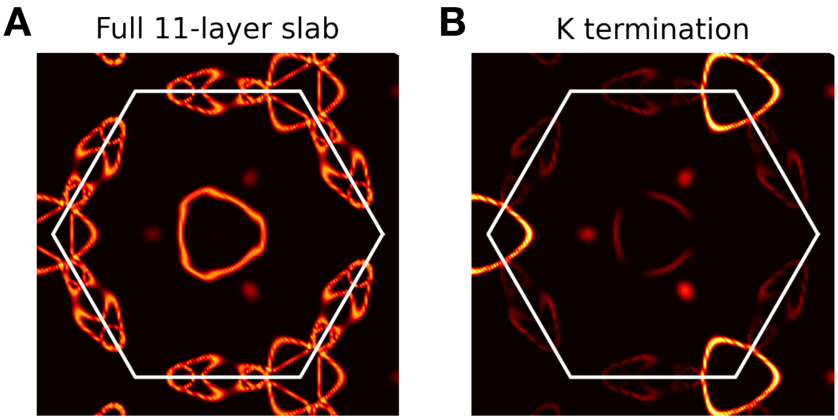

Fig. S8: **Spectral function for $U = 1.3$ eV, 11 layers EIO**. The spectral function is computed as (**A**) including the full slab and (**B**) projected onto the kagome termination.

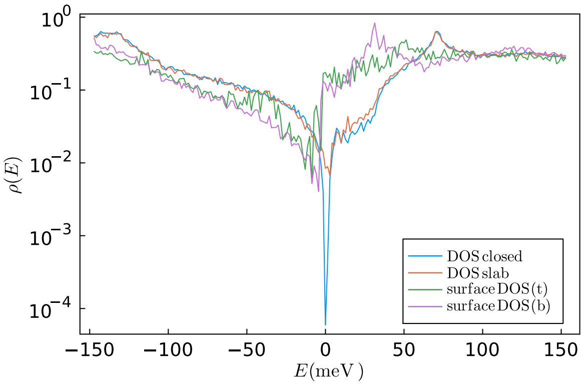

Fig. S9: **The density of states (DOS) comparison between slab and closed geometries.** The surface DOS are obtained by projecting into surface sites. "t" and "b" denote the top and bottom surfaces. Results are obtained with $L_x = L_y = 10$, $L_z = 61$ and KPM expansion order $N_C = 2^{17}$.

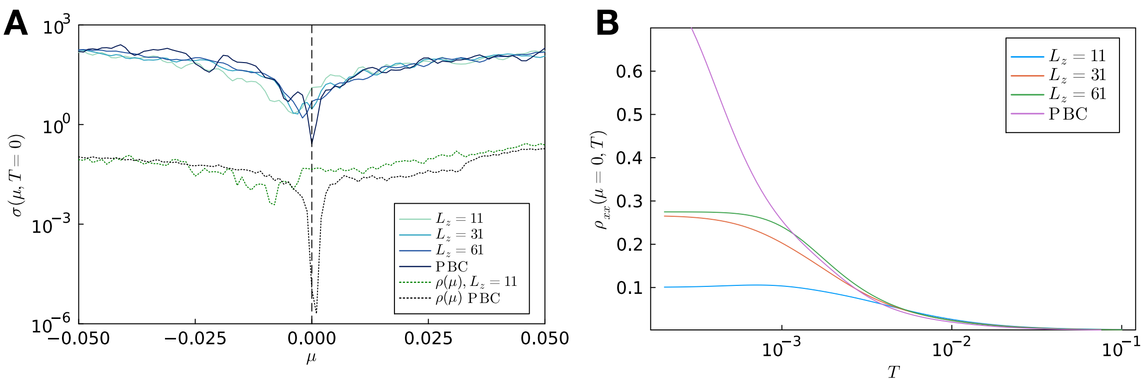

Fig. S10: **The longitudinal conductivity and resistivity around the semimetallic Fermi level.** (**A**) The longitudinal conductivity at zero temperature as a function of Fermi energies. The KPM expansion order $N_C = 2^{13}$ for the conductivity calculation. Dotted curves are the bulk DOS results for comparison with $N_C = 2^{17}$. (**B**) the corresponding resistivity $\rho_{xx} \approx 1/\sigma_{xx}$ as a function temperature.

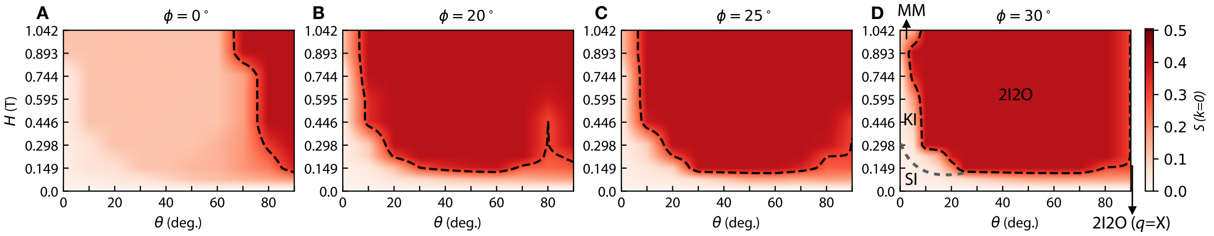

Fig. S11: **The computed $S(\mathbf{k}=0)$ for all the spins in one kagome surface**. The computation includs all three sublattices in the simulated bulk DTO, with a magnetic field tilted away from [111], along the directions of $\phi = 0°$, $20°$, $25°$, and $30°$, which correspond to panels **A-D**, respectively. The direction of $\phi = 0°$ corresponds to DTO's 2I2O zero-momentum phase, and $30°$ ([1$\bar{1}$0]) the finite-momentum phase if the magnetic field is in-plane ($\theta = 90°$). For $\phi \geq 20°$, DTO shows a SI-KI-MM transition with small $\theta \leq 20°$, while the large region in the phase diagram shows only 2I2O spin ice phases.

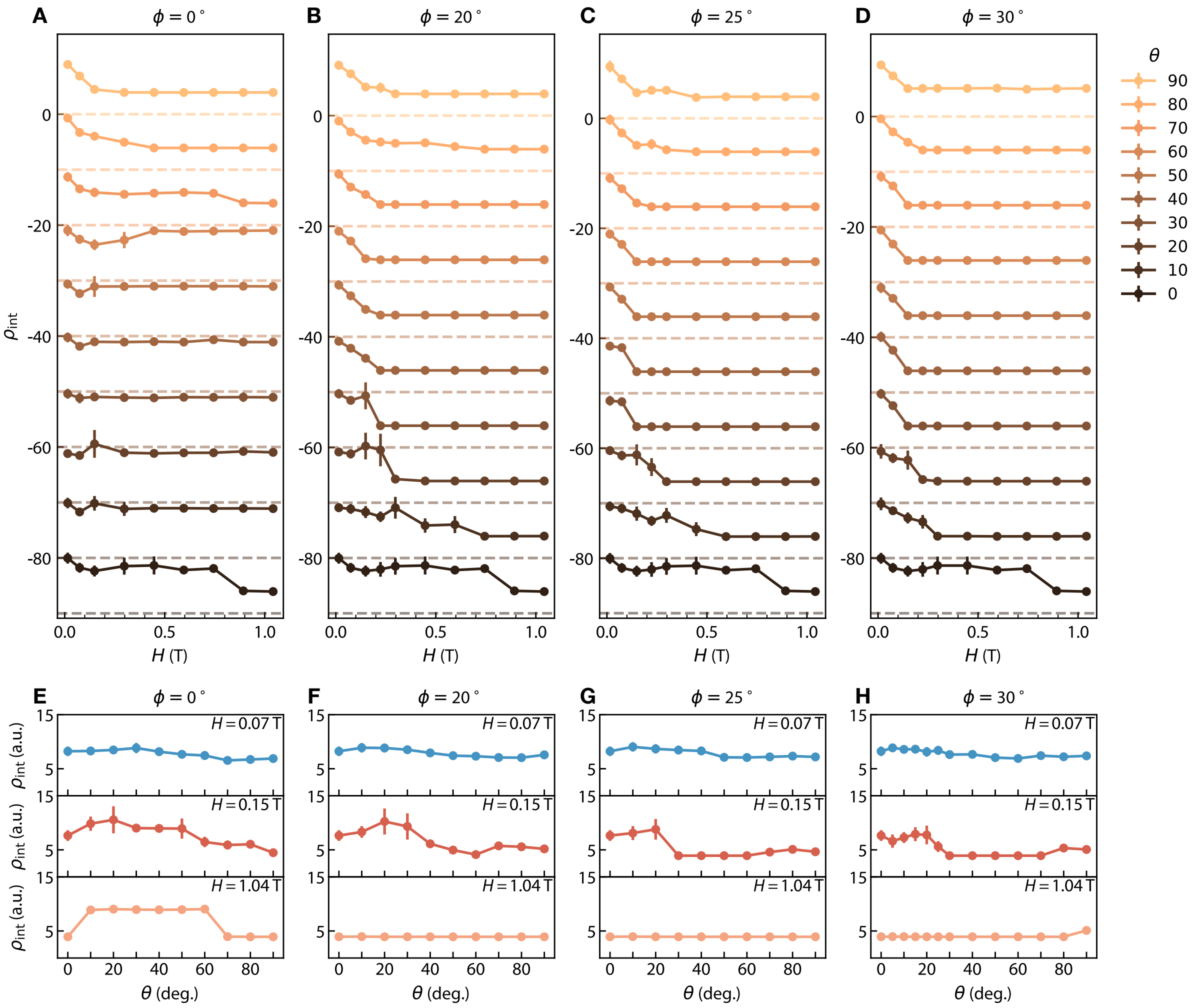

Fig. S12: **The computed ρ_{int} for magnetic field tilted away from [111]**. The computation includes the directions of $\phi = 0°$ (panels **A,E**), $20°$ (panels **B,F**), $25°$ (panels **C,G**), and $30°$ (panels **D,H**). The direction of $\phi = 0°$ corresponds to DTO's 2I2O zero-momentum phase, and $30°$ ($[[1\bar{1}0]]$) the finite-momentum phase if the magnetic field is in-plane ($\theta = 90°$). The lower four panels show the same data for the three chosen magnetic fields. We can see that, at the onset of the kagome ice phase (0.15 T), the interfacial resistivity ρ_{int} shows a local maximum near $\theta = 20°$. This angular anisotropy in ρ_{int} agrees qualitatively with the observed angular-dependent MR shown in Fig. 2B and 2C.

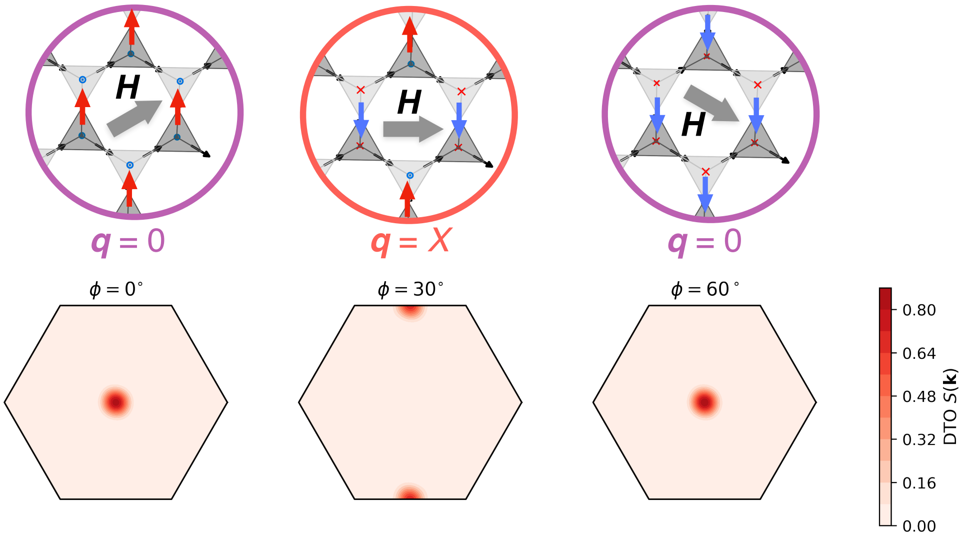

Fig. S13: **DTO magnetic phases under three field directions.** Upper panels: The MC averaged snapshots at $H = 0.6\,\mathrm{T}$ (in-plane rotating magnetic field). Lower panels: computed $S(\mathbf{k})$ for the spins in one kagome surface in the simulated bulk DTO (only the spins in the relevant sublattice are taken into account, as highlighted in the upper panels by the red and blue in-plane arrows). $\phi = 0°$ and $60°$ correspond to DTO's 2I2O zero-momentum phase, while $30°$ (when the magnetic field is perpendicular to all the spins in this sublattice) corresponds to a finite-momentum ($q = X$) phase, therefore showing maxima in $S(\mathbf{k})$ at the Brillouin zone boundaries.

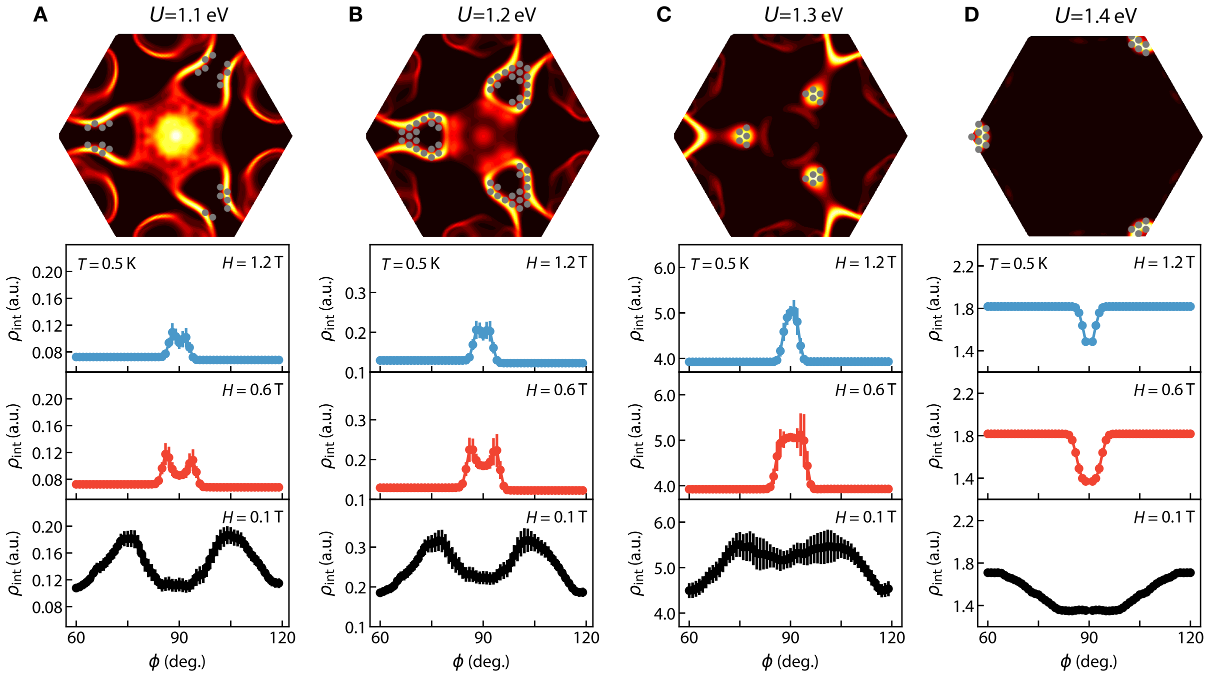

Fig. S14: **Computed surface spectral function of EIO and the interfacial resistivity under different U.** Computed surface spectral function of EIO projected onto kagome terminations, and the interfacial resistivity due to the presence of the interfacial Kondo coupling, with an in-plane rotating magnetic field, for the metallic surface states of EIO modeled by DFT+U downfolded tight-binding models. Panels (**A**)-(**D**) correspond to U=1.1, 1.2, 1.3, 1.4 eV. The grey dots represent the momenta $\mathbf{k}_{\mathrm{F}}$'s that have been integrated over on a sparse 36×36 uniform k-grid. As U increases from 1.1 eV to 1.4 eV, EIO always shows 24 Weyl cones. However, the kagome-termination projected spectral function shows very different patterns, suggesting that the topological surface states have distinct momenta as U varies. Since Eq. 15 are dominated by large $S(\mathbf{q})$ (zone-center for zero-momentum phase, and zone-boundary for finite-momentum phase), and the projected Weyl node positions, tuned by U, determine the scattering vectors $\mathbf{q}$'s, one would expect that the resultant ρ_{int} depends on U as well.

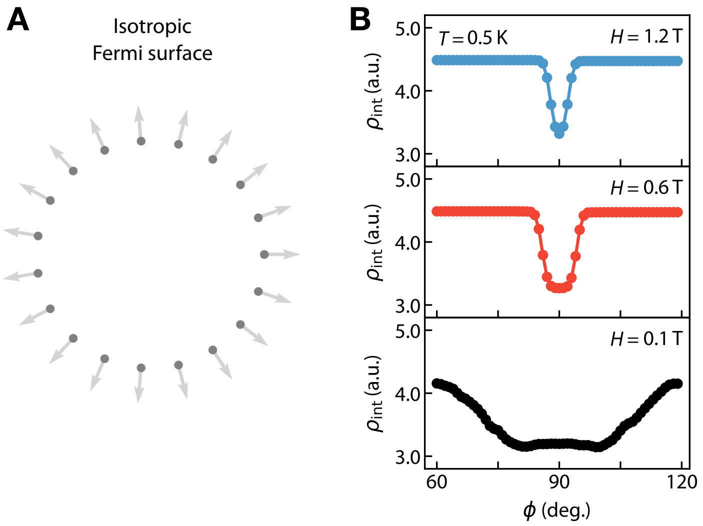

Fig. S15: **Consideration of isotropic 2D Fermi surface.** (**A**) A manually constructed isotropic 2D Fermi surface. The grey dots are the sampled k points on the Fermi surface, and the arrows show the direction of Fermi velocity at each point. This Fermi surface could represent the Fermi surface of a 2D electron gas. (**B**) Computed ρ_{int} response using the Fermi surface shown in (A). The minima of ρ_{int} occur at finite-momentum phase.

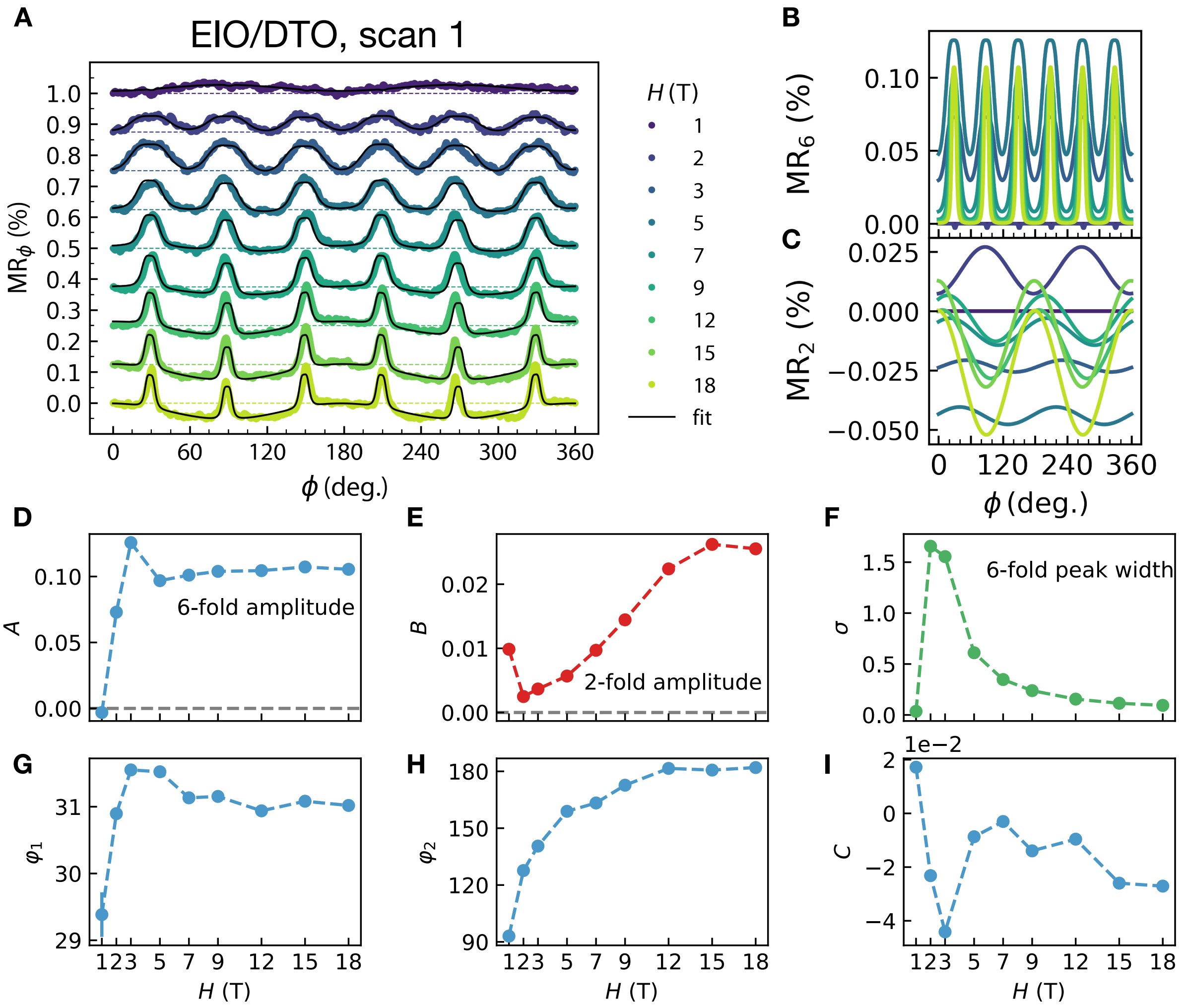

Fig. S16: **A natïve fit of the in-plane measured magnetoresistance.** A natïve fit of the in-plane measured magnetoresistance MR_ϕ for the data set obtained with scan 1 using Eq. 17. (**A**) MR_ϕ given by the transport measurement of the EIO/DTO sample, with an in-plane rotating magnetic field. The black curves are the results predicted by fitting to Eq. 17. (**B** to **C**) the six-fold and the left-over two-fold anisotropic components MR_6 and MR_2. One can see that MR_6 narrows as the field strength increases, and the two-fold anisotropy is large at 1 T and also at high fields. (**D** to **I**) Fitted parameters six-fold amplitude A, two-fold amplitude B (also shown in the top panel in the main text Fig. 3C, labeled as $|\mathrm{MR}_6|$ and $|\mathrm{MR}_2|$), six-fold peaks width σ, phases φ_1 and φ_2 (also shown in lower panel of Fig. 3C), and constant C. We can see from (E) that the two-fold anisotropy is always present in our naïve fit and from (F) that the six-fold peaks become narrower as the field strength increases. Meanwhile, (H) shows that the phase φ_2 of the two-fold anisotropy changes from $90°$ to $180°$ continuously as the magnetic field increases from 1 T to 18 T.

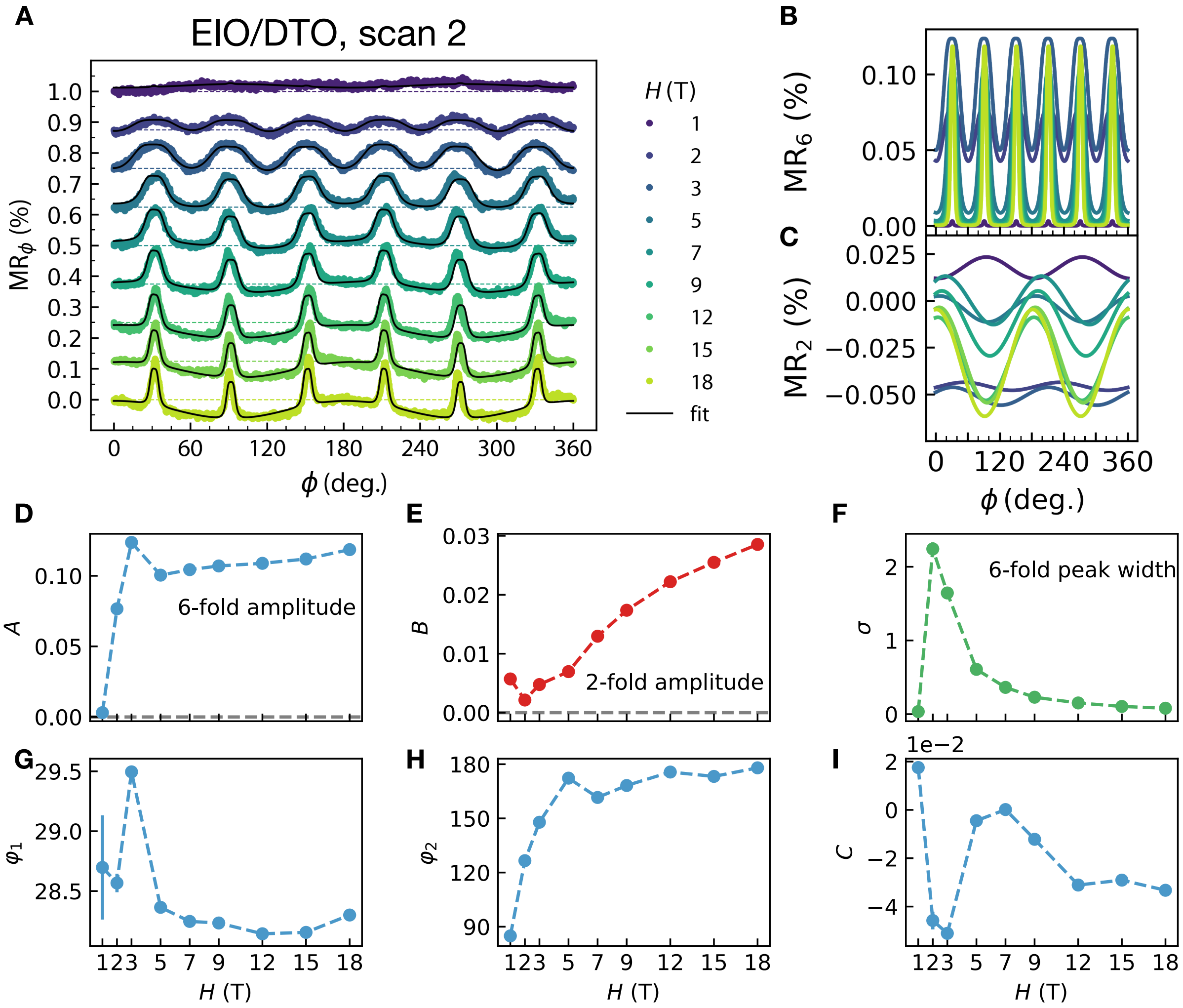

Fig. S17: **A natïve fit of the in-plane measured magnetoresistance for scan 2.** Same analysis as Fig. S16, but for scan 2's data. (**A**) MR_ϕ given by the transport measurement of the EIO/DTO sample, with an in-plane rotating magnetic field. The black curves are the results predicted by fitting to Eq. 17. (**B** to **C**) the six-fold and the left-over two-fold anisotropic components MR_6 and MR_2. One can see that MR_6 narrows as the field strength increases, and the two-fold anisotropy is large at 1 T and also at high fields. (**D** to **I**) Fitted parameters six-fold amplitude A, two-fold amplitude B (also shown in the top panel in the main text Fig. 3C, labeled as $|MR_6|$ and $|MR_2|$), six-fold peaks width σ, phases φ_1 and φ_2 (also shown in lower panel of Fig. 3C), and constant C. We can see from (E) that the two-fold anisotropy is always present in our naïve fit and from (F) that the six-fold peaks become narrower as the field strength increases. Meanwhile, (H) shows that the phase φ_2 of the two-fold anisotropy changes from $90°$ to $180°$ continuously as the magnetic field increases from 1 T to 18 T.

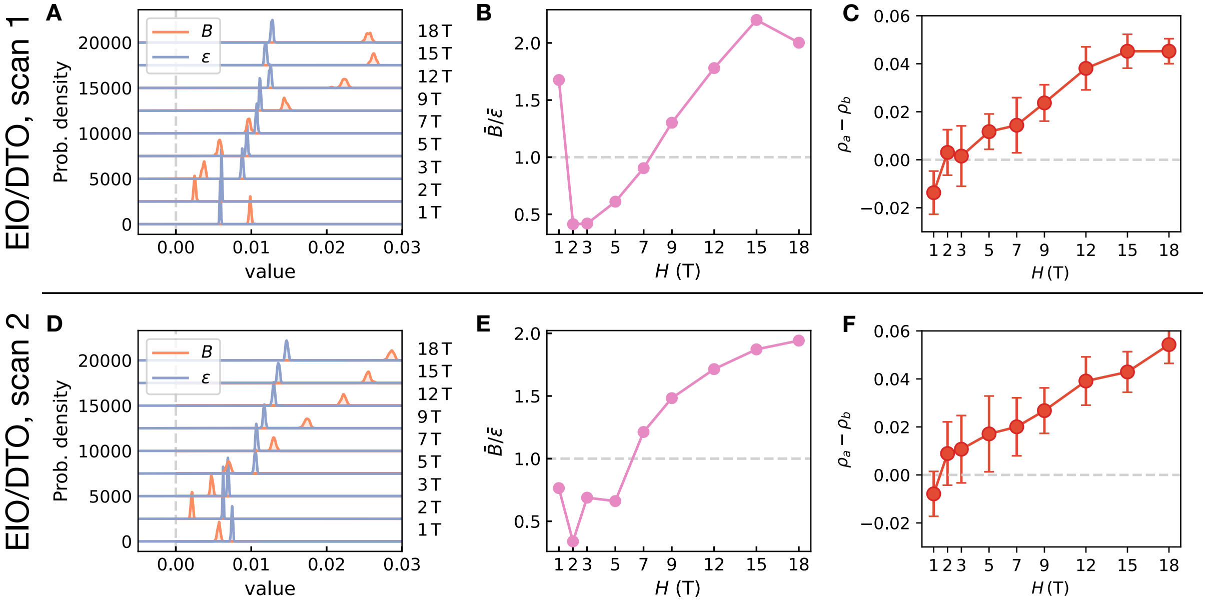

Fig. S18: **Bayesian inference fitting parameters.** Bayesian inference of the parameter B and its error ϵ assuming that our model (Eq. 17) describes the experimental data, compared with "ovality" that measures the two-fold anisotropy in MR_ϕ, obtained for EIO/DTO, scan 1 (upper 3 panels) and 2 (lower 3 panels). (**A** and **D**) The probability distribution of the two-fold anisotropy amplitude B and its error ϵ in Eq. 18. (**B** and **E**) The ratio between the mean values of B and ϵ, $\bar{B}/\bar{\epsilon}$, versus the magnetic field H. One can say that the 2-fold anisotropy emerges when $\bar{B}/\bar{\epsilon} > 1$. Therefore, the predicted H_{c2} is likely to be between 7 and 9 T. This is qualitatively consistent with the other measurement we defined in the main text, ovality, which measures direct the 2-fold anisotropy strength from the raw data, and gives the transition field H_{c2} to be between 3 and 5 T. (**C** and **F**) Computed "ovality" $(\rho_a - \rho_b)$, with $\rho_a = [\rho(180^\circ) + \rho(360^\circ)]/2$ and $\rho_b = [\rho(60^\circ) + \rho(120^\circ) + \rho(240^\circ) + \rho(300^\circ)]/4$. This quantity measures the two-fold anisotropy in MR_ϕ, and is nonzero within error bars for $H = 1\,\mathrm{T}$, and $H \geq 5\,\mathrm{T}$, qualitatively consistent with the results given by Bayesian inference analysis.

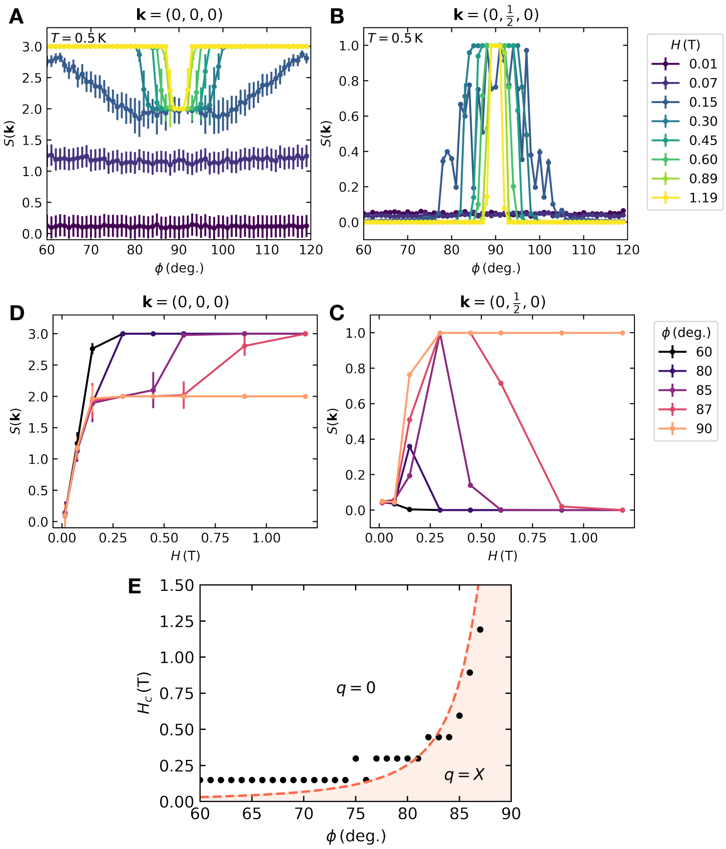

Fig. S19: **Calculated spin structure and phase diagram of DTO.** (**A to D**) The spin structure factor $S(\mathbf{k})$ at momenta $(0, 0, 0)$ (panels A and C) and $(0, \frac{1}{2}, 0)$ (panels B and D), computed from Monte Carlo simulatiron for the bulk DTO with $8 \times 8 \times 2$ cell size, summed over three sublattices, with in-plane magnetic fields ranging from 0.01 to 1.19 T. Panels A and B show the values of $S(\mathbf{k})$ as a function of the rotation angle. Panel C and D show the same values but as a function of the field strength, for 5 different rotation angles, which indicates how the critical field strength increases as ϕ approaches $90°$. (**E**) The schematic phase diagram of the bulk spin ice given by Monte Carlo simulation. The phase boundary is estimated as the field strength when $S(X)$ drops below 0.02 for each angle ϕ. The red dashed line is a guide to the eye and is expected to be subject to the size effect.

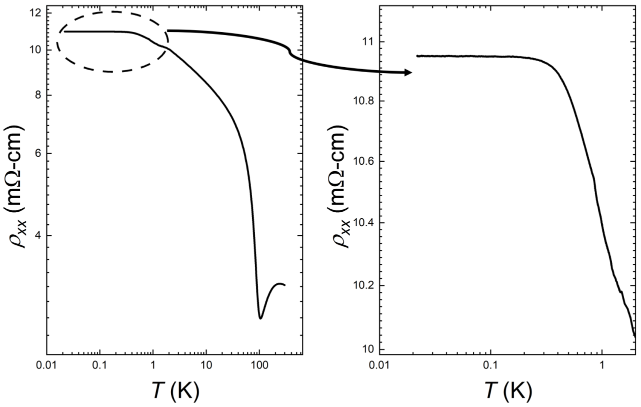

Fig. S20: **Temperature-dependent resistivity of EIO/DTO.** The left figure shows the entire temperature range from 20 mK to 300 K and the right figure shows the zoomed-in view from 20 mK to 2 K. The data are the same as Fig. 1C of the manuscript.

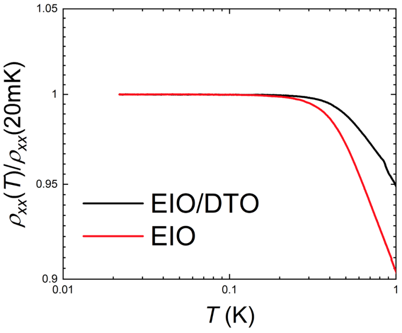

Fig. S21: **Temperature-dependent resistivity of EIO/DTO and EIO at low temperatures.** Temperature-dependent resistivity of EIO/DTO (black) and EIO (red) below 1 K. The data are the same as Fig. 1C of the manuscript.

REFERENCES AND NOTES

1. P. Zubko, S. Gariglio, M. Gabay, P. Ghosez, J.-M. Triscone, Interface physics in complex oxide heterostructures. *Annu. Rev. Condens. Matter Phys.* **2**, 141–165 (2011).

2. H. Y. Hwang, Y. Iwasa, M. Kawasaki, B. Keimer, N. Nagaosa, Y. Tokura, Emergent phenomena at oxide interfaces. *Nat. Mater.* **11**, 103–113 (2012).

3. J. Chakhalian, J. W. Freeland, A. J. Millis, C. Panagopoulos, J. M. Rondinelli, Colloquium: Emergent properties in plane view: Strong correlations at oxide interfaces. *Rev. Mod. Phys.* **86**, 1189–1202 (2014).

4. R. Ramesh, D. G. Schlom, Creating emergent phenomena in oxide superlattices. *Nat. Rev. Mater.* **4**, 257–268 (2019).

5. R. Bistritzer, A. H. MacDonald, Moiré bands in twisted double-layer graphene. *Proc. Natl. Acad. Sci. U.S.A.* **108**, 12233–12237 (2011).

6. L. Balents, C. R. Dean, D. K. Efetov, A. F. Young, Superconductivity and strong correlations in moiré flat bands. *Nat. Phys.* **16**, 725–733 (2020).

7. E. Y. Andrei, D. K. Efetov, P. Jarillo-Herrero, A. H. MacDonald, K. F. Mak, T. Senthil, E. Tutuc, A. Yazdani, A. F. Young, The marvels of moiré materials. *Nat. Rev. Mater.* **6**, 201–206 (2021).

8. M. Yankowitz, Q. Ma, P. Jarillo-Herrero, B. J. LeRoy, van der Waals heterostructures combining graphene and hexagonal boron nitride. *Nat. Rev. Phys.* **1**, 112–125 (2019).

9. D. M. Kennes, M. Claassen, L. Xian, A. Georges, A. J. Millis, J. Hone, C. R. Dean, D. N. Basov, A. N. Pasupathy, A. Rubio, Moiré heterostructures as a condensed-matter quantum simulator. *Nat. Phys.* **17**, 155–163 (2021).

10. Y. Cao, V. Fatemi, S. Fang, K. Watanabe, T. Taniguchi, E. Kaxiras, P. Jarillo-Herrero, Unconventional superconductivity in magic-angle graphene superlattices. *Nature* **556**, 43–50 (2018).

11. M. Gibertini, M. Koperski, A. F. Morpurgo, K. S. Novoselov, Magnetic 2D materials and heterostructures. *Nat. Nanotechnol.* **14**, 408–419 (2019).

12. W. Li, X. Qian, J. Li, Phase transitions in 2D materials. *Nat. Rev. Mater.* **6**, 829–846 (2021).

13. A. Ciarrocchi, F. Tagarelli, A. Avsar, A. Kis, Excitonic devices with van der Waals heterostructures: Valleytronics meets twistronics. *Nat. Rev. Mater.* **7**, 449–464 (2022).

14. B. Huang, M. A. McGuire, A. F. May, D. Xiao, P. Jarillo-Herrero, X. Xu, Emergent phenomena and proximity effects in two-dimensional magnets and heterostructures. *Nat. Mater.* **19**, 1276–1289 (2020).

15. L. Du, T. Hasan, A. Castellanos-Gomez, G.-B. Liu, Y. Yao, C. N. Lau, Z. Sun, Engineering symmetry breaking in 2D layered materials. *Nat. Rev. Phys.* **3**, 193–206 (2021).

16. K. F. Mak, D. Xiao, J. Shan, Light–valley interactions in 2D semiconductors. *Nat. Photonics* **12**, 451–460 (2018).

17. S. Y. F. Zhao, X. Cui, P. A. Volkov, H. Yoo, S. Lee, J. A. Gardener, A. J. Akey, R. Engelke, Y. Ronen, R. Zhong, G. Gu, S. Plugge, T. Tummuru, M. Kim, M. Franz, J. H. Pixley, N. Poccia, P. Kim, Time-reversal symmetry breaking superconductivity between twisted cuprate superconductors. *Science* **382**, 1422–1427 (2023).

18. M. Martini, Y. Lee, T. Confalone, S. Shokri, C. N. Saggau, D. Wolf, G. Gu, K. Watanabe, T. Taniguchi, D. Montemurro, V. M. Vinokur, K. Nielsch, N. Poccia, Twisted cuprate van der Waals heterostructures with controlled Josephson coupling. *Mater. Today* **67**, 106–112 (2023).

19. Y. Lee, M. Martini, T. Confalone, S. Shokri, C. N. Saggau, D. Wolf, G. Gu, K. Watanabe, T. Taniguchi, D. Montemurro, V. M. Vinokur, K. Nielsch, N. Poccia, Encapsulating high-temperature superconducting twisted van der waals heterostructures blocks detrimental effects of disorder. *Adv. Mater.* **35**, 2209135 (2023).

20. J. Chakhalian, X. Liu, G. A. Fiete, Strongly correlated and topological states in [111] grown transition metal oxide thin films and heterostructures. *APL Mater.* **8**, 050904 (2020).

21. J. S. Gardner, M. J. P. Gingras, J. E. Greedan, Magnetic pyrochlore oxides. *Rev. Mod. Phys.* **82**, 53–107 (2010).

22. J. G. Rau, M. J. P. Gingras, Frustrated quantum rare-earth pyrochlores. *Annu. Rev. Condens. Matter Phys.* **10**, 357–386 (2019).

23. W. Witczak-Krempa, G. Chen, Y. B. Kim, L. Balents, Correlated quantum phenomena in the strong spin-orbit regime. *Annu. Rev. Condens. Matter Phys.* **5**, 57–82 (2014).

24. M. Ohno, T. C. Fujita, M. Kawasaki, Proximity effect of emergent field from spin ice in an oxide heterostructure. *Sci. Adv.* **10**, eadk6308 (2024).

25. J.-H. She, C. H. Kim, C. J. Fennie, M. J. Lawler, E.-A. Kim, Topological superconductivity in metal/quantum-spin-ice heterostructures. *npj Quantum Mater.* **2**, 64 (2017).

26. L. Miao, Y. Lee, A. B. Mei, M. J. Lawler, K. M. Shen, Two-dimensional magnetic monopole gas in an oxide heterostructure. *Nat. Commun.* **11**, 1341 (2020).

27. H. Zhang, C. Xing, K. Noordhoek, Z. Liu, T. Zhao, L. Horák, Q. Huang, L. Hao, J. Yang, S. Pandey, E. Dagotto, Z. Jiang, J. H. Chu, Y. Xin, E. S. Choi, H. Zhou, J. Liu, Anomalous magnetoresistance by breaking ice rule in $Bi_2Ir_2O_7/Dy_2Ti_2O_7$ heterostructure. *Nat. Commun.* **14**, 1404 (2023).

28. X. Wan, A. M. Turner, A. Vishwanath, S. Y. Savrasov, Topological semimetal and Fermi-arc surface states in the electronic structure of pyrochlore iridates. *Phys. Rev. B* **83**, 205101 (2011).

29. M. Udagawa, L. Jaubert, *Spin Ice* (Springer, 2021).

30. M. J. Gingras, "Spin ice" in *Introduction to Frustrated Magnetism: Materials, Experiments, Theory* (Springer, 2010), pp. 293–329.

31. S. T. Bramwell, M. J. Gingras, Spin ice state in frustrated magnetic pyrochlore materials. *Science* **294**, 1495–1501 (2001).

32. C. Castelnovo, R. Moessner, S. L. Sondhi, Spin ice, fractionalization, and topological order. *Annu. Rev. Condens. Matter Phys.* **3**, 35–55 (2012).

33. S. T. Bramwell, M. J. Harris, The history of spin ice. *J. Phys. Condens. Matter* **32**, 374010 (2020).

34. G.-W. Chern, R. Moessner, Dipolar order by disorder in the classical heisenberg antiferromagnet on the kagome lattice. *Phys. Rev. Lett.* **110**, 077201 (2013).

35. M. Kareev, X. Liu, M. Terilli, F. Wen, T.-C. Wu, D. Doughty, H. Li, J. Zhou, Q. Zhang, L. Gu, J. Chakhalian, Epitaxial stabilization of a pyrochlore interface between Weyl semimetal and spin ice. *Nano Lett.* **25**, 966–972 (2025).

36. F. Wen, T.-C. Wu, X. Liu, M. Terilli, M. Kareev, J. Chakhalian, Epitaxial stabilization of (111)-oriented frustrated quantum pyrochlore thin films. *J. Appl. Phys.* **129**, 025302 (2021).

37. X. Liu, S. Fang, Y. Fu, W. Ge, M. Kareev, J.-W. Kim, Y. Choi, E. Karapetrova, Q. Zhang, L. Gu, E.-S. Choi, F. Wen, J. H. Wilson, G. Fabbris, P. J. Ryan, J. W. Freeland, D. Haskel, W. Wu, J. H. Pixley, J. Chakhalian, Magnetic Weyl semimetallic phase in thin films of $Eu_2Ir_2O_7$. *Phys. Rev. Lett.* **127**, 277204 (2021).

38. G. Chen, M. Hermele, Magnetic orders and topological phases from *f*-*d* exchange in pyrochlore iridates. *Phys. Rev. B* **86**, 235129 (2012).

39. B.-J. Yang, N. Nagaosa, Emergent topological phenomena in thin films of pyrochlore iridates. *Phys. Rev. Lett.* **112**, 246402 (2014).

40. X. Liu, J.-W. Kim, Y. Wang, M. Terilli, X. Jia, M. Kareev, S. Peng, F. Wen, T.-C. Wu, H. Chen, W. Hu, M. H. Upton, J. Kim, Y. Choi, D. Haskel, H. Weng, P. J. Ryan, Y. Cao, Y. Qi, J. Guo, J. Chakhalian, Chiral spin-liquid-like state in pyrochlore iridate thin films. *Nat. Commun.* **15**, 10348 (2024).

41. G. Resta, S.-T. Pi, X. Wan, S. Y. Savrasov, High surface conductivity of Fermi-arc electrons in Weyl semimetals. *Phys. Rev. B* **97**, 085142 (2018).

42. M. Breitkreiz, P. W. Brouwer, Large contribution of Fermi arcs to the conductivity of topological metals. *Phys. Rev. Lett.* **123**, 066804 (2019).

43. C. Zhang, Y. Zhang, H.-Z. Lu, X. C. Xie, F. Xiu, Cycling Fermi arc electrons with Weyl orbits. *Nat. Rev. Phys.* **3**, 660–670 (2021).

44. F. Buccheri, A. De Martino, R. G. Pereira, P. W. Brouwer, R. Egger, Phonon-limited transport and Fermi arc lifetime in Weyl semimetals. *Phys. Rev. B* **105**, 085410 (2022).

45. Z. Xiang, K.-W. Chen, L. Chen, T. Asaba, Y. Sato, N. Zhang, D. Zhang, Y. Kasahara, F. Iga, W. A. Coniglio, Y. Matsuda, J. Singleton, L. Li, Hall anomaly, quantum oscillations and possible Lifshitz transitions in Kondo insulator YbB_{12}: Evidence for unconventional charge transport. *Phys. Rev. X* **12**, 021050 (2022).

46. Z. Ren, A. A. Taskin, S. Sasaki, K. Segawa, Y. Ando, Large bulk resistivity and surface quantum oscillations in the topological insulator Bi_2Te_2Se. *Phys. Rev. B* **82**, 241306 (2010).

47. S. Jia, H. Beidenkopf, I. Drozdov, M. K. Fuccillo, J. Seo, J. Xiong, N. P. Ong, A. Yazdani, R. J. Cava, Defects and high bulk resistivities in the Bi-rich tetradymite topological insulator $Bi_{2+x}Te_{2-x}Se$. *Phys. Rev. B* **86**, 165119 (2012).

48. D.-J. Kim, J. Xia, Z. Fisk, Topological surface state in the Kondo insulator samarium hexaboride. *Nat. Mater.* **13**, 466–470 (2014).

49. M. Dzero, K. Sun, V. Galitski, P. Coleman, Topological Kondo insulators. *Phys. Rev. Lett.* **104**, 106408 (2010).

50. F. Tafti, Q. Gibson, S. K. Kushwaha, N. Haldolaarachchige, R. J. Cava, Resistivity plateau and extreme magnetoresistance in LaSb. *Nat. Phys.* **12**, 272–277 (2016).

51. B. S. Tan, Y.-T. Hsu, B. Zeng, M. C. Hatnean, N. Harrison, Z. Zhu, M. Hartstein, M. Kiourlappou, A. Srivastava, M. D. Johannes, T. P. Murphy, J.-H. Park, L. Balicas, G. G. Lonzarich, G. Balakrishnan, S. E. Sebastian, Unconventional Fermi surface in an insulating state. *Science* **349**, 287–290 (2015).

52. L. Li, K. Sun, C. Kurdak, J. W. Allen, Emergent mystery in the Kondo insulator samarium hexaboride. *Nat. Rev. Phys.* **2**, 463–479 (2020).

53. T. Liang, J. Lin, Q. Gibson, S. Kushwaha, M. Liu, W. Wang, H. Xiong, J. A. Sobota, M. Hashimoto, P. S. Kirchmann, Z.-X. Shen, R. J. Cava, N. P. Ong, Anomalous Hall effect in $ZrTe_5$. *Nat. Phys.* **14**, 451–455 (2018).

54. S.-Y. Xu, I. Belopolski, N. Alidoust, M. Neupane, G. Bian, C. Zhang, R. Sankar, G. Chang, Z. Yuan, C.-C. Lee, S.-M. Huang, H. Zheng, J. Ma, D. S. Sanchez, B. K. Wang, A. Bansil, F. Chou, P. P. Shibayev, H. Lin, S. Jia, M. Z. Hasan, Discovery of a Weyl fermion semimetal and topological Fermi arcs. *Science* **349**, 613–617 (2015).

55. P. J. W. Moll, N. L. Nair, T. Helm, A. C. Potter, I. Kimchi, A. Vishwanath, J. G. Analytis, Transport evidence for Fermi-arc-mediated chirality transfer in the dirac semimetal Cd_3As_2. *Nature* **535**, 266–270 (2016).

56. A. G. Grushin, J. W. F. Venderbos, A. Vishwanath, R. Ilan, Inhomogeneous Weyl and dirac semimetals: Transport in axial magnetic fields and Fermi arc surface states from pseudo-landau levels. *Phys. Rev. X* **6**, 041046 (2016).

57. M. Z. Hasan, S.-Y. Xu, I. Belopolski, S.-M. Huang, Discovery of Weyl fermion semimetals and topological Fermi arc states. *Annu. Rev. Condens. Matter Physi.* **8**, 289–309 (2017).

58. A. A. Zyuzin, A. A. Burkov, Topological response in Weyl semimetals and the chiral anomaly. *Phys. Rev. B* **86**, 115133 (2012).

59. J. Xiong, S. K. Kushwaha, T. Liang, J. W. Krizan, M. Hirschberger, W. Wang, R. J. Cava, N. P. Ong, Evidence for the chiral anomaly in the Dirac semimetal Na_3Bi. *Science* **350**, 413–416 (2015).

60. R. Higashinaka, Y. Maeno, Field-induced transition on a triangular plane in the spin-ice compound $Dy_2Ti_2O_7$. *Phys. Rev. Lett.* **95**, 237208 (2005).

61. H. Sato, K. Matsuhira, T. Tayama, Z. Hiroi, S. Takagi, T. Sakakibara, Ferromagnetic ordering on the triangular lattice in the pyrochlore spin-ice compound $Dy_2Ti_2O_7$. *J. Phys. Condens. Matter* **18**, L297–L303 (2006).

62. K. Matsuhira, H. Sato, T. Tayama, Z. Hiroi, S. Takagi, T. Sakakibara, Observation of a novel phase transition induced by a magnetic field in the pyrochlore spin ice compound. *J. Phys. Condens. Matter* **19**, 145269 (2007).

63. W.-H. Kao, P. C. W. Holdsworth, Y.-J. Kao, Field-induced ordering in dipolar spin ice. *Phys. Rev. B* **93**, 180410 (2016).

64. T. Suzuki, L. Savary, J.-P. Liu, J. W. Lynn, L. Balents, J. G. Checkelsky, Singular angular magnetoresistance in a magnetic nodal semimetal. *Science* **365**, 377–381 (2019).

65. J. Seo, C. De, H. Ha, J. E. Lee, S. Park, J. Park, Y. Skourski, E. S. Choi, B. Kim, G. Y. Cho, H. W. Yeom, S.-W. Cheong, J. H. Kim, B.-J. Yang, K. Kim, J. S. Kim, Colossal angular magnetoresistance in ferrimagnetic nodal-line semiconductors. *Nature* **599**, 576–581 (2021).

66. J. P. C. Ruff, R. G. Melko, M. J. P. Gingras, Finite-temperature transitions in dipolar spin ice in a large magnetic field. *Phys. Rev. Lett.* **95**, 097202 (2005).

67. Z. Wang, K. Barros, G.-W. Chern, D. L. Maslov, C. D. Batista, Resistivity minimum in highly frustrated itinerant magnets. *Phys. Rev. Lett.* **117**, 206601 (2016).

68. L. Bovo, X. Moya, D. Prabhakaran, Y.-A. Soh, A. T. Boothroyd, N. D. Mathur, G. Aeppli, S. T. Bramwell, Restoration of the third law in spin ice thin films. *Nat. Commun.* **5**, 3439 (2014).

69. L. D. C. Jaubert, T. Lin, T. S. Opel, P. C. W. Holdsworth, M. J. P. Gingras, Spin ice thin film: Surface ordering, emergent square ice, and strain effects. *Phys. Rev. Lett.* **118**, 207206 (2017).

70. L. Bovo, C. M. Rouleau, D. Prabhakaran, S. T. Bramwell, Layer-by-layer epitaxial thin films of the pyrochlore $Tb_2Ti_2O_7$. *Nanotechnology* **28**, 055708 (2017).

71. L. Bovo, C. M. Rouleau, D. Prabhakaran, S. T. Bramwell, Phase transitions in few-monolayer spin ice films. *Nat. Commun.* **10**, 1219 (2019).

72.. Lantagne-Hurtubise, J. G. Rau, M. J. Gingras, Spin-ice thin films: Large-*N* theory and Monte Carlo simulations. *Phys. Rev. X* **8**, 021053 (2018).

73. K. Barry, B. Zhang, N. Anand, Y. Xin, A. Vailionis, J. Neu, C. Heikes, C. Cochran, H. Zhou, Y. Qiu, W. Ratcliff, T. Siegrist, C. Beekman, Modification of spin-ice physics in $Ho_2Ti_2O_7$ thin films. *Phys. Rev. Mater.* **3**, 084412 (2019).

74. D. P. Leusink, F. Coneri, M. Hoek, S. Turner, H. Idrissi, G. Van Tendeloo, H. Hilgenkamp, Thin films of the spin ice compound $Ho_2Ti_2O_7$. *APL Mater.* **2**, 032101 (2014).

75. J. P. Clancy, J. P. C. Ruff, S. R. Dunsiger, Y. Zhao, H. A. Dabkowska, J. S. Gardner, Y. Qiu, J. R. D. Copley, T. Jenkins, B. D. Gaulin, Revisiting static and dynamic spin-ice correlations in $Ho_2Ti_2O_7$ with neutron scattering. *Phys. Rev. B* **79**, 014408 (2009).

76. N. Shannon, O. Sikora, F. Pollmann, K. Penc, P. Fulde, Quantum ice: A quantum Monte Carlo study. *Phys. Rev. Lett.* **108**, 067204 (2012).

77. K. A. Ross, L. Savary, B. D. Gaulin, L. Balents, Quantum excitations in quantum spin ice. *Phys. Rev. X* **1**, 021002 (2011).

78. J. S. Gardner, S. R. Dunsiger, B. D. Gaulin, M. J. P. Gingras, J. E. Greedan, R. F. Kiefl, M. D. Lumsden, W. A. MacFarlane, N. P. Raju, J. E. Sonier, I. Swainson, Z. Tun, Cooperative paramagnetism in the geometrically frustrated pyrochlore antiferromagnet $Tb_2Ti_2O_7$. *Phys. Rev. Lett.* **82**, 1012–1015 (1999).

79. J. S. Gardner, B. D. Gaulin, A. J. Berlinsky, P. Waldron, S. R. Dunsiger, N. P. Raju, J. E. Greedan, Neutron scattering studies of the cooperative paramagnet pyrochlore $Tb_2Ti_2O_7$. *Phys. Rev. B* **64**, 224416 (2001).

80. T. A. Bojesen, S. Onoda, Quantum spin ice under a [111] magnetic field: From pyrochlore to kagome. *Phys. Rev. Lett.* **119**, 227204 (2017).

81. A. M. Hallas, J. Gaudet, B. D. Gaulin, Experimental insights into ground-state selection of quantum XY pyrochlores. *Annu. Rev. Condens. Matter Phys.* **9**, 105–124 (2018).

82. P. Goswami, S. Chakravarty, Quantum criticality between topological and band insulators in 3 + 1 dimensions. *Phys. Rev. Lett.* **107**, 196803 (2011).

83. A. A. Burkov, L. Balents, Weyl semimetal in a topological insulator multilayer. *Phys. Rev. Lett.* **107**, 127205 (2011).

84. A. Weiße, G. Wellein, A. Alvermann, H. Fehske, The kernel polynomial method. *Rev. Mod. Phys.* **78**, 275–306 (2006).

85. J. H. García, L. Covaci, T. G. Rappoport, Real-space calculation of the conductivity tensor for disordered topological matter. *Phys. Rev. Lett.* **114**, 116602 (2015).

86. S. M. João, J. V. P. Lopes, Basis-independent spectral methods for non-linear optical response in arbitrary tight-binding models. *J. Phys. Condens. Matter* **32**, 125901 (2020).

87. A.-K. Wu, D. Guerci, Y. Fu, J. H. Wilson, J. H. Pixley, Absence of quantization in the circular photogalvanic effect in disordered chiral Weyl semimetals. *Phys. Rev. B* **110**, 014201 (2024).

88. B. C. den Hertog, M. J. P. Gingras, Dipolar interactions and origin of spin ice in ising pyrochlore magnets. *Phys. Rev. Lett.* **84**, 3430–3433 (2000).

89. G. D. Mahan, *Many-Particle Physics* (Springer Science & Business Media, 2013).

90. O. Abril-Pla, V. Andreani, C. Carroll, L. Dong, C. J. Fonnesbeck, M. Kochurov, R. Kumar, J. Lao, C. C. Luhmann, O. A. Martin, M. Osthege, R. Vieira, T. Wiecki, R. Zinkov, PyMC: A modern and comprehensive probabilistic programming framework in python. *PeerJ Comput. Sci.* **9**, e1516 (2023).

91. D. Qvarngård, P. Henelius, Classical spin models and basic magnetic interactions on 1/1-approximant crystals. *Phys. Rev. B* **110**, 104414 (2024).

92. H. Wang, V. Harbola, Y.-J. Wu, P. A. van Aken, J. Mannhart, Interface design beyond epitaxy: Oxide heterostructures comprising symmetry-forbidden interfaces. *Adv. Mater.* **36**, e2405065 (2024).
